# Influence of Liquid Crystallinity and Mechanical Deformation on the Molecular Relaxations of an Auxetic Liquid Crystal Elastomer

**DOI:** 10.3390/molecules26237313

**Published:** 2021-12-02

**Authors:** Thomas Raistrick, Matthew Reynolds, Helen F. Gleeson, Johan Mattsson

**Affiliations:** School of Physics and Astronomy, University of Leeds, Leeds LS2 9JT, UK; py14tr@leeds.ac.uk (T.R.); M.Reynolds@leeds.ac.uk (M.R.); h.f.gleeson@leeds.ac.uk (H.F.G.)

**Keywords:** liquid crystalline elastomer, dielectric spectroscopy, shear rheology, polymer relaxations

## Abstract

Liquid Crystal Elastomers (LCEs) combine the anisotropic ordering of liquid crystals with the elastic properties of elastomers, providing unique physical properties, such as stimuli responsiveness and a recently discovered molecular auxetic response. Here, we determine how the molecular relaxation dynamics in an acrylate LCE are affected by its phase using broadband dielectric relaxation spectroscopy, calorimetry and rheology. Our LCE is an excellent model system since it exhibits a molecular auxetic response in its nematic state, and chemically identical nematic or isotropic samples can be prepared by cross-linking. We find that the glass transition temperatures ($T_{g}$) and dynamic fragilities are similar in both phases, and the T-dependence of the α relaxation shows a crossover at the same $T^{*}$ for both phases. However, for $T>T^{*}$, the behavior becomes Arrhenius for the nematic LCE, but only more Arrhenius-like for the isotropic sample. We provide evidence that the latter behavior is related to the existence of pre-transitional nematic fluctuations in the isotropic LCE, which are locked in by polymerization. The role of applied strain on the relaxation dynamics and mechanical response of the LCE is investigated; this is particularly important since the molecular auxetic response is linked to a mechanical Fréedericksz transition that is not fully understood. We demonstrate that the complex Young’s modulus and the α relaxation time remain relatively unchanged for small deformations, whereas for strains for which the auxetic response is achieved, significant increases are observed. We suggest that the observed molecular auxetic response is coupled to the strain-induced out-of-plane rotation of the mesogen units, in turn driven by the increasing constraints on polymer configurations, as reflected in increasing elastic moduli and α relaxation times; this is consistent with our recent results showing that the auxetic response coincides with the emergence of biaxial order.

## 1. Introduction

Liquid Crystal Elastomers (LCEs) are lightly cross-linked polymer networks with mesogen units incorporated within the main polymer chain, or as pendant units. LCEs combine the anisotropic behavior of liquid crystals, arising from the long-range orientational order of the mesogen units, with the rubber-like elastic behavior of conventional elastomers [1]. The macroscopic shape of LCEs is coupled to the ordering of the mesogen units making them stimuli response materials [2,3]. LCEs can also show a wide range of other useful properties such as stress–optical coupling [4], soft elastic deformation [5], biocompatibility [6] and enhanced damping properties [7].

To understand, and be able to predict material behavior, it is essential to understand the behavior of the relevant molecular or segmental relaxations. Examples include the links between relaxations and material aging and rejuvenation [8], the response to mechanical stress of polymer glasses [9,10,11,12], or the link between structural relaxation and ionic transport in polymer electrolytes [13]. Due to the unique properties of LCEs, including their often complex mechanical responses [5,7,14], it is important to characterize their relaxation dynamics. Furthermore, the relaxation dynamics and glass-formation in nematic liquid crystals in general is of significant fundamental interest [15,16,17], and LCEs allow for careful investigations of the behavior in nematic materials over a wide temperature range. The LCE in our study is particularly important in this regard, as it exhibits a molecular auxetic response [18,19] and chemically identical samples can be formed, by polymerization, in either the nematic or the isotropic phase. The existence of the nematic or isotropic phase for our LCE system has been demonstrated using Raman spectroscopy, where scalar order parameter ($S=1/2\langle 3\cos^{2}\theta -1\rangle$) values of 0.59 ± 0.05 and 0.00 ± 0.05 were found, respectively [4,19]. In addition, polarized optical microscopy and Berek compensator measurements confirm these results [4,20]. Moreover, differential scanning calorimetry (DSC) experiments show no evidence for any nematic-to-isotropic phase transition over the investigated temperature ranges, thus confirming that the phase is locked-in during synthesis [4,20]. Thus, the effects of the nematic order on glass formation and molecular relaxation can be directly, and elegantly, probed for a system of identical chemical composition. Furthermore, by determining the response on both rheology and relaxation dynamics to strain, offers important insight into the nature of the molecular auxetic behavior.

The structural (α) relaxation of a glass-forming liquid (or polymer melt), slows down dramatically upon cooling. If crystallization is avoided, e.g., by fast cooling, the material eventually falls out of equilibrium, resulting in a disordered solid—a glass. The glass transition temperature, $T_{g}$, is typically defined as the temperature (T) for which the characteristic α relaxation time, $\tau_{\alpha }$, reaches 100 s [21]. Upon cooling towards the glassy state, the molecular motions involved become increasingly cooperative [22,23], typically involving a few hundred molecules (or polymer segments) at $T_{g}$ even though the detailed behavior is system-dependent [24]. In addition to the α-relaxation, glass-forming liquids or polymers typically show at least one additional secondary molecular relaxation that is generic to glass-formation and is linked to the α relaxation [25,26,27]; this is typically termed the β relaxation or the Johari–Goldstein β relaxation [28]. The β relaxation separates from the structural α relaxation below a temperature ~$T_{\alpha \beta }$, persists in the glassy state, and since the glass structure is largely frozen (disregarding slow physical aging), its behavior can be characterized by a single fixed activation energy and is thus well-described by an Arrhenius expression:

(1) $$\tau_{\beta }=\tau_{0}\exp\left(\frac{\Delta E_{A}}{k_{B}T}\right).$$

Here, $\tau_{0}$ is a microscopic relaxation time ($∼10^{-13}$ s), $\Delta E_{A}$ is the activation energy and $k_{B}$ is the Boltzmann constant. The α relaxation, on the other hand, typically shows a more complex non-Arrhenius temperature dependence with a T-dependent activation energy that grows for decreasing T. Empirically $\tau_{\alpha }\left(T\right)$ is often described using a so-called Vogel–Fulcher–Tammann (VFT) equation [21,22]:

(2) $$\tau_{\alpha }=\tau_{0}\exp\left(\frac{DT_{0}}{T-T_{0}}\right),$$

where $\tau_{0}$ is a microscopic relaxation time, $T_{0}$ is the temperature at which $\tau_{\alpha }$ tends to infinity and D is a parameter which controls the extent of deviation of $\tau_{\alpha }$ from Arrhenius behavior, the so-called ‘fragility’; an alternative commonly used metric of fragility is $m=\mathrm{dlog}(\tau_{\alpha })/d\left(T_{g}/T\right)|T=T_{g}$. ‘Fragile’ liquids are highly sensitive to a T-change near $T_{g}$ and are thus characterized by large m, or conversely, small D-values. In contrast, ‘strong’ liquids are characterized by small m, or large D-values [29]. Typically, strong liquids show near Arrhenius $\tau_{\alpha }\left(T\right)$ behaviour, whereas fragile liquids are highly non-Arrhenius [21,23,29]. An empirical VFT function can often describe the $\tau_{\alpha }\left(T\right)$ behavior well over an extended T-range above $T_{g}$. However, for temperature above *T*$∼T_{B}$, where $T_{B}∼\;1.2-1.6\times T$_g_ (the $T_{B}/T_{g}$ ratio is system-dependent and has shown a systematic variation with fragility [30]), the T-dependence often crosses over into another VFT-like behavior, with a more significant non-Arrhenius *T*-dependence [31]. This dynamic crossover, at $T_{B}$, generally coincides relatively well with $T_{\alpha ,\beta }$ [25,27] and with several other changes in the liquid including a decoupling of translational and rotational diffusion [21] and a change in the T-dependence of the α-relaxation strength [22]. Furthermore, the relaxation behavior for $T>T_{B}$ is often relatively well-described by so-called ideal-Mode Coupling Theory (ideal MCT) [22], but the correspondence with its predictions break down for $T∼T_{B}$. Thus, $T_{B}$ signifies a fundamental change in the liquid dynamics, which is an observation predicted already in the 1960s by Goldstein [32] and associated with changes in molecular relaxations due to the need to overcome energy barriers within the experienced ‘energy landscape’ that become significantly larger than $k_{B}T$ for *T*$<T_{B}$. Finally, at high T, above a temperature $T_{A}$ ($T_{A}>T_{B}>T_{g}$), the need for cooperative motions in the α relaxation disappears, or is significantly reduced, resulting in Arrhenius behavior with a fixed energy barrier [31].

The general glass-transition phenomenology for polymeric glass-formers is similar to that of non-polymeric systems. However, due to chain connectivity and the corresponding presence of intra-molecular degrees of freedom, the inter-relation between the α relaxation and the secondary relaxations is more complicated [33,34]. Moreover, for sufficiently long oligomers (longer than a Kuhn or Rouse bead) or polymers, the difference between the $\tau_{\alpha }\left(T\right)$ behaviour on either side of $T_{B}$ is often reduced, or disappear altogether [24,35], meaning that only one effective VFT is observed across a wide T-range; the origin of this behavior is not presently well understood. Additionally, for polymers, the transition to Arrhenius behavior at high-T is often difficult to study due to polymer degradation [23]. Cross-linked polymer systems, in turn, show similar relaxation behavior to other polymeric glass-forming materials. However, the presence and density of cross-links can affect the relaxation behavior, and the effects on the structural α-relaxation are typically to slow it down, resulting in increased $T_{g}$ [36].

For LCEs, there have been relatively few studies focusing on molecular relaxation behavior [37,38,39]. The nomenclature for glass-transition-related relaxation dynamics of LCEs typically follows that used for side-chain liquid crystal polymers (SCLCPs). In SCLCPs, 4 relaxation processes are typically observed: δ, α, β and γ, named in order of increasing relaxation frequency for a fixed T [22]. The structural (α) relaxation in SCLCPs involves the backbone polymer segments and is directly related to the glass transition [22,40]. The δ process has been observed in both SCLCPs [22,41,42,43] and LCEs [37,38,39] and is typically slower than the α relaxation, follows a VFT dependence, and is attributed to reorientation of the mesogenic units around the polymer backbone. The faster secondary relaxations, typically termed β and γ are generally assigned to motions of the mesogenic units, where the β relaxation is typically assigned to fluctuations of the mesogen around its molecular long axis [22,41,44,45], and the γ relaxation is assigned to motions of either the spacer unit, or the terminal group of the side-chain mesogen [40,41]. 

In SCLCPs, just as for non-LC glass formers, a cross-over in dynamic behavior has been observed for $\tau_{\alpha }\left(T\right)$. However, in contrast to the non-LC behavior, for SCLCPs the low-T VFT behavior typically changes to a higher T Arrhenius behavior at a crossover temperature, $T^{*\;}∼1.1-1.3\times T_{g}$ [42,46]. Interestingly, $T^{*}/T_{g}$ is similar to $T_{B}/T_{g},$ as observed for non-LC systems, suggesting similarities in their origins. Moreover, this cross-over to Arrhenius behavior is typically only observed for polymers that form LC phases. For example, in one study, the existence of LC phase behavior was removed from a SCLCP by the substitution of a hydrogen on the biphenyl mesogen group with the bulkier methoxy (-OCH3) group. This resulted in a loss of the crossover from VFT to Arrhenius at $T^{*}$, and $\tau_{\alpha }$ could instead be described by a single VFT [42]. This observation suggests that fluctuations related to LC phases are required for this behavior to occur. 

It is important to understand how LCEs respond to applied deformations. LCEs are typically characterized by two different types of response to an applied strain. The first is the Semi-Soft Elastic (SSE) response [5,47]. Here, the elastic cost of deformation is reduced by the continuous rotation of the nematic director in counter-rotating domains known as ‘stripe domains’ [47]. In the semi-soft elastic response, it is assumed that the nematic order of the system remains constant whilst the orientation of the director is free to rotate in response to the applied strain [1,47]. Thus, for SSE LCEs, it has been shown that the shear storage modulus perpendicular to the director is smaller than parallel to the director, $G_{∥}^{\prime }>G_{⊥}^{\prime }$ [48,49]; this ‘softening’ is believed to be due to the rotation of the director. A number of investigations have been performed to determine the rheological behavior and also the relaxation behavior for typical LCEs that undergo SSE. Examples include LCEs based on siloxane [37,38,48,49,50] or acrylate [39] chemistry, with a side-chain mesogenic attachment. The second class of LCEs deforms via a completely different mechanism known as the mechanical Fréedericksz transition [19,20,51]. Here, a discontinuous rotation of the director is observed upon application of strain, as opposed to the continuous rotation of SSE. Before the discontinuous rotation occurs, the director is essentially fixed and only the degree of nematic order changes with applied strain [1,52]. Additionally, in some of the LCEs that deform via a mechanical Fréedericksz transition, a *negative* Poisson ratio in one of the transverse axes is observed [18], and this behavior has been identified as a molecular auxetic response [18,19]. LCEs that deform via the mechanical Fréedericksz transition, which include the LCE of this study, typically have an acrylate backbone [20,51]. The underlying mechanism that causes an LCE to deform via a discontinuous rotation instead of displaying the SSE response is still to be determined, and the theoretical framework behind the two mechanisms appear to be different [1]. To address this and better understand the LCEs of the second class, we here perform a detailed characterization of both the molecular relaxations and rheological behavior for an acrylate-based LCE which is previously known to deform via the mechanical Fréedericksz transition. Additionally, we investigate how both applied strain and the liquid crystal phase affects the molecular relaxation dynamics and the rheological response of an all-acrylate LCE.

## 2. Results and Discussion

### 2.1. Relaxation Dynamics of the Isotropic and Nematic LCE

**Figure 1:**
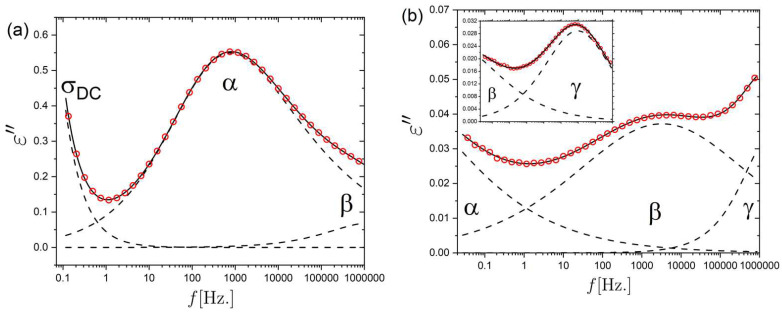
Dielectric loss versus frequency for the nematic LCE sample. The fits to the data, described in the main text, are shown in solid lines, and the individual contributions from the α, β and γ relaxations, as well as the DC conductivity ($\sigma_{DC})$ are labelled and shown in dashed lines. Data for different temperatures are shown in: (**a**) T= 315.15 K, (**b**) main graph: T= 248.15 K and inset: 163.15 K.

Broadband Dielectric Spectroscopy (BDS) was performed for both an unstrained nematic and isotropic LCE sample, and the complex dielectric permittivity $\varepsilon^{*}\left(f\right)=\varepsilon^{\prime }\left(f\right)-i\varepsilon^{\prime \prime }\left(f\right)$ was determined over a wide frequency range (~10^−2^–10^6^ Hz) (for details see the Section 3). Results for the dielectric loss, $\varepsilon^{\prime \prime }\left(f\right)$, of the nematic LCE are shown in Figure 1 for a few selected temperatures; the dielectric response over the full temperature range is included in the Supplementary Materials (SM) (Figure S1). For both LCE samples, three relaxation processes are identified, where α denotes the structural relaxation, directly related to the glass-transition, and β and γ, corresponding to more local motions, are characterized by shorter timescales. In addition, a clear contribution from ionic DC-conductivity is present, as evidenced by the power law contribution $\varepsilon^{\prime \prime }\propto f$^−1^ observed at low frequencies in Figure 1a.

LCEs previously investigated in literature also typically show a so-called δ relaxation that is slower than the α relaxation and associated with the motions of the mesogenic unit around its short axis [37,38,39]. To investigate whether there is evidence for any dielectrically active molecular relaxation slower than the α relaxation, in the dynamic range where DC-conductivity dominates the dielectric response, we use two additional approaches: (i) We estimate the dielectric loss free of conductivity effects $\varepsilon_{der}^{\prime \prime }$ by conversion from the $\varepsilon^{\prime }$ spectra using a well-established simple approximation of the Kramers–Kronig transformation [53]:

(3) $$\varepsilon_{der}^{\prime \prime }\left(\omega \right)=-\frac{\pi }{2}\frac{\partial \varepsilon^{\prime }\left(\omega \right)}{\partial \ln\omega }\;\mathrm{where}\;\omega =2\pi f\;.$$

A pure DC-conductivity is only observed in the dielectric loss, $\varepsilon^{\prime \prime }$, and not in $\varepsilon^{\prime }$; thus, this approach can significantly reduce the interference from DC-conductivity in the analysis. (ii) We analyzed data in the dielectric modulus representation $M^{*}=1/\varepsilon^{*}$, for which the DC-conductivity contribution is generally suppressed [53]. For both these approaches, a slow Debye-like relaxation was identified in the dynamic window slower than the α relaxation (see Supplementary Materials). This Debye-relaxation coincides with the presence of electrode polarization, as observed by a low-frequency increase in $\varepsilon^{\prime }$. We thus interpret this relaxation peak as arising from so-called conductivity relaxation [43,53], due to electrode polarization, and thus not to a molecular relaxation; electrode polarization is due to charge accumulation at the sample-electrode interface [22]. Given the interference from DC-conductivity and electrode polarization at low frequencies, we cannot completely rule out that a slow δ process exists. However, we do not find any evidence for it within the investigated temperature and frequency range. LCEs, previously studied in the literature, that display a δ relaxation, also show a transition from an isotropic phase to an LC phase. Conversely, the LCE investigated here shows no such transition [4,20], nor is there evidence for the δ relaxation, as discussed above; This suggests that despite the presence of side-chain mesogenic units, the larger-scale motions of the mesogenic units are hindered in both the isotropic and nematic phases of the LCE. The cross-link density of our LCE, based on chemical composition, is 7.1 mol%. This cross-linking density is comparable to the 7.5 mol% cross-link density in a previous study of an LCE with a comparable $T_{g}$ value, which did show a δ relaxation [38], suggesting that the level of crosslinking in our LCE might not be enough to trap the mesogen large-scale movements. Thus, we instead suggest that the presence of the non-mesogen pendant units (EHA; see MS) in our system, could entrap the mesogen A6OCB sidechain, in turn preventing the larger-scale motions corresponding to the δ− relaxation.

**Figure 2:**
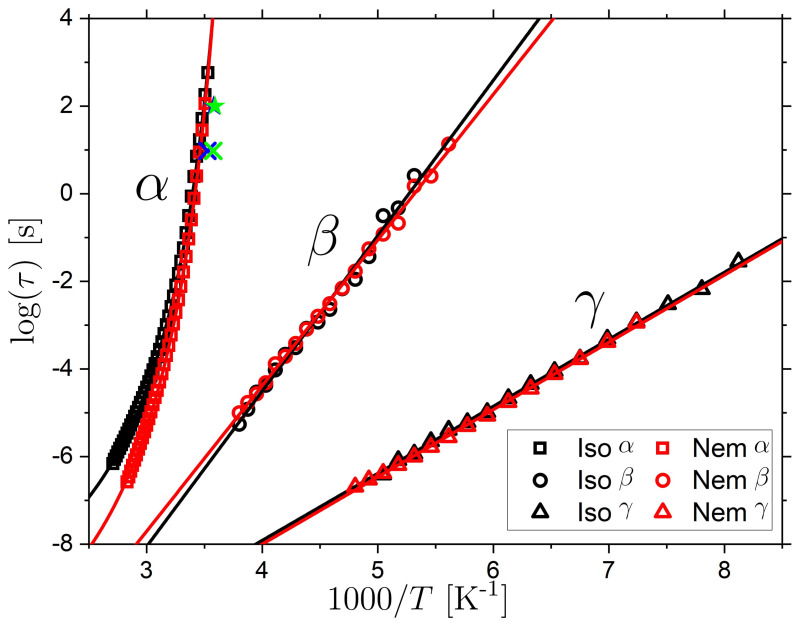
Characteristic relaxation times for the α (squares), β (circles) and γ (triangles) relaxations identified for the isotropic (black) and nematic (red) LCE samples. The results of a VFT fit to the α relaxation data, and Arrhenius fits the β and γ relaxation data are shown in solid lines. Data for the glass transition temperature (α relaxations) determined from modulated DSC (stars) are also shown as corresponding to a time-scale of $\tau_{0}=$ 9.56 s, for the isotropic (blue) and nematic (green) LCE sample. Glass-transition temperature data using DSC performed at a fixed rate of 10 °C/min (crosses) are plotted assuming a corresponding time-scale of $\tau_{\alpha }$ = 100 s (see text for further discussion).

To investigate the *T*-dependent complex permittivity $\varepsilon^{*}\left(f\right)$, the data are fit by a sum of relaxation contributions and a contribution for the DC-conductivity (see Section 3). The α relaxation is fit with a Havriliak–Negami (HN) expression:

(4) $$\varepsilon^{*}\left(f\right)=\varepsilon_{\infty }+\frac{\Delta \varepsilon }{{\left(1+{\left(i2\pi f\tau_{\mathrm{HN}}\right)}^{p}\right)}^{q}}\;$$

where $\varepsilon_{\infty }$ is the high-frequency permittivity, Δε is the dielectric strength, $\tau_{\mathrm{HN}}$ is the HN characteristic timescale, p  and q are shape parameters of the response function; p corresponds to the low-frequency power law exponent and p×q, the high-frequency exponent of the relaxation. The α relaxation was found to have similar broadness and asymmetry (based on p and p×q values) in the isotropic and nematic LCE. The T range for which the α relaxation is in the probed frequency window is ~290 K to 360 K. As an example, at 319 K the HN parameters characterizing the α relaxation are p≈0.5 and p×q≈0.2 for both the isotropic and nematic LCE. The secondary β and γ relaxations were well-described by the simpler Cole–Cole expression, for which q was set to 1 in Equation (4); this leads to a relaxation peak that is symmetric on a logarithmic frequency axis. The β relaxation was found to have a fairly constant value of p, with mean values 0.21±0.01 and 0.25±0.01 for the isotropic and nematic phases, respectively. The p value for the γ relaxation was found to increase in a linear fashion from ~0.25 to ~0.4 for increasing temperature. We choose the most probable relaxation time, corresponding to the peak maximum, as the characteristic timescale for each relaxation contribution; The peak relaxation times are defined as $\tau_{p}={\left(2\pi f_{p}\right)}^{-1}$ where $\tau_{p}$ is the frequency corresponding to the peak maximum. For the HN function, which is generally asymmetric (i.e., q≠0), the HN timescale is not the timescale of the peak maximum, but $\tau_{p}$ can be derived from $\tau_{\mathrm{HN}}$,

p

and q (see Section 3). The T-dependent characteristic relaxation times for the α, β and γ relaxations are shown in an Arrhenius plot in Figure 2. The α relaxations for the isotropic (Iso) and nematic (Nem) LCE samples are here fitted using a VFT expression, whereas the β and γ relaxations are fitted using an Arrhenius expression. The fitting parameters resulting from the fits are provided in Table 1. 

As seen from the fits in Figure 2 and the corresponding parameters, the β and γ relaxations have very similar T-dependencies in the isotropic and nematic states. The corresponding activation energies $\Delta E_{A}$ for the β relaxation are $67.9\;{\mathrm{kJ}\;\mathrm{mol}}^{-1}$ and $63.6\;{\mathrm{kJ}\;\mathrm{mol}}^{-1}$ for the isotropic and nematic LCE, respectively. The corresponding $\Delta E_{A}$ results for the γ relaxation are $29.3{\;\mathrm{kJ}\;\mathrm{mol}}^{-1}$ and $29.4\;{\mathrm{kJ}\;\mathrm{mol}}^{-1},\;\mathrm{respectively}$. The results of the Arrhenius fits thus demonstrate that the LCE phase has little effect on the β and γ relaxations. This may be due to the relatively short characteristic length scales of these relaxations, in comparison to the relevant length scale of the LC phase. It is interesting to compare our LCE results to those of other LC systems in the literature. A collection of activations energies, $\Delta E_{A}$, for β relaxations in polyacrylate and polymethacrylate SCLCPs are found in work by Kremer and Schönhals [22] and Schönhals and Hans-eckartcarius [40], $\Delta E_{A}$ values within the range $46.5–68.9\;{\mathrm{kJ}\;\mathrm{mol}}^{-1}$ were reported. The exact $\Delta E_{A}$ value depends on the terminal group attachment of the mesogenic unit and the length of the alkyl spacer between the backbone and the mesogenic unit. Our acrylate-based LCE has a spacer length of 6. The activation energy of the SCLCP with the closest chemistry to our LCE (acrylate backbone, spacer length of 6) is 62.8 ${\mathrm{kJ}\;\mathrm{mol}}^{-1}$, which is close to our $\Delta E_{A}$ values of $67.9\;{\mathrm{kJ}\;\mathrm{mol}}^{-1}$ and $63.6\;{\mathrm{kJ}\;\mathrm{mol}}^{-1}$ for the isotropic and nematic LCE respectively. Thus, we follow the literature assignment and associate the observed β relaxation with fluctuations of the mesogen around its long axis. Literature values of the γ relaxation, typically assigned to motions of the alkyl spacer units, have $\Delta E_{A}$ values in the range $\sim 33-35\;{\mathrm{kJ}\;\mathrm{mol}}^{-1}$ [41,54,55], which is close to the $\Delta E_{A}$ of ~29 ${\;\mathrm{kJ}\;\mathrm{mol}}^{-1}$ determined for the γ relaxation in the isotropic and nematic LCE. Hence, based on comparison to literature data, the γ process for our LCE is likely due to motions of the 6-alkyl chain connecting A6OCB to the acrylate backbone. 

The characteristic timescale of the α-relaxation, $\tau_{\alpha \;}\left(T\right)$, follows a non-Arrhenius T-dependence in both the isotropic and nematic phase, and we use an empirical VFT expression to describe the behavior (Table 1). However, as discussed in the introduction, for molecular glass formers the T-dependence of the α relaxation can often not be described accurately using a single VFT equation. Thus, to further investigate the detailed T-dependence of $\tau_{\alpha }\left(T\right)$ we perform a derivative analysis of the data, as first suggested by Stickel et al. [31,56]. By plotting the parameter Z vs. 1000/T where Z is given by:

(5) $$Z={\left(\frac{d\log\tau_{\alpha }}{d\left(\frac{1000}{T}\right)}\right)}^{-\frac{1}{2}},$$

a VFT-behavior is linearized and this analysis has been shown to be useful in identifying changes in the T-dependence [31,56]. 

**Figure 3:**
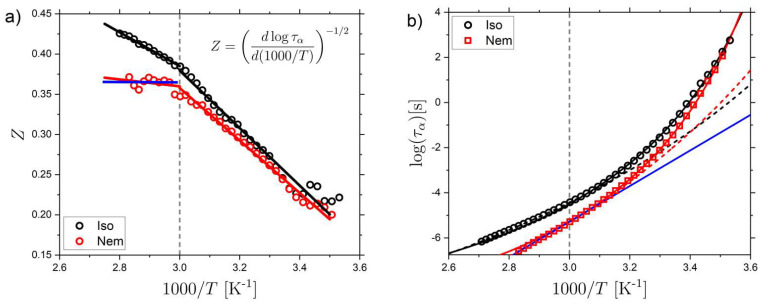
(**a**) Derivative analysis of $\tau_{\alpha }\left(T\right)$ data (see text for details) for the isotropic (black) and nematic (red) LCE samples, respectively. A crossover behavior is observed at $T^{*}\approx$ 333 K, as shown with a dashed line. Linear fits of the data are shown both for temperatures above and below $T^{*}$. For the nematic samples the data for $T>T^{*}$ can be well described using a horizontal line (corresponding to Arrhenius behavior). (**b**) Arrhenius plot of $\tau_{\alpha }\left(T\right)$ vs. inverse temperature for the isotropic and nematic samples. For $T<T^{*}$, the VFT fits to the data are shown in solid lines. For $T>T^{*\;}$ VFT fits are shown in dashed lines for the isotropic (black) and nematic (red) sample. An Arrhenius fit is also applied to the nematic data before 1000/T=3.0 (blue solid line).

As shown in Figure 3a, both the isotropic and nematic LCE samples undergo a change in $\tau_{\alpha }\left(T\right)$ at a temperature $T^{*}\approx$ 333 K, corresponding to $1000/T^{*}\approx 3.0$. For $T<T^{*}$, the gradients of the linear fits are similar (−0.36 and −0.33 for the isotropic and nematic samples, respectively). However, for $T>T^{*}$, the gradients are clearly significantly different (−0.21 and −0.04). A clear change in $\tau_{\alpha }\left(T\right)$ for both the isotropic and nematic samples are therefore supported by the derivative analysis, as evidenced by a change in gradient at $T^{*}$. Moreover, it is clear that the gradient for the nematic samples for $T>T^{*}$ is very near zero and can thus be well described by an Arrhenius behavior. To further investigate $\tau_{\alpha }\left(T\right)$, the data are fit with separate VFTs, for $T<T^{*}$ (solid lines) and $T>T^{*}$ (dashed lines), as shown in Figure 3b. For the nematic data, for $T>T^{*}$, the data were also fit using an Arrhenius expression for comparison. The results of the fits are outlined in Table 2.

From the fit result of the dielectric data, the $T_{g}$ values can be determined from $T_{g}=T\left(\tau_{\alpha }=100\;s\right)$, which results in $T_{g}$ values of 286 K and 285 K, respectively. These $T_{g}\;$ values can be compared with those obtained from m-DSC and DSC experiments. (see Supplementary Materials for DSC traces) The DSC measurements, performed on cooling at 10 K/min, give $T_{g}$ values of 279 K and 278 K, which is relatively close to $T_{g}$ from BDS. The modulation period of m-DSC (60 s) probes the α relaxation on a timescale of ~10 s (see Section 3 for full details). The values of $T_{g}$ from m-DSC are 283 K and 279 K for isotropic and nematic LCE. Thus, the trend where $\tau_{\alpha }\left(T\right)\left[\mathrm{iso}\right]>\tau_{\alpha }\left(T\right)\left[\mathrm{nem}\right]$ for $T>T_{g}$ is confirmed by the m-DSC measurements. Furthermore, the similar VFT parameters D=5.4 and D=5.1 reflect that $\tau_{\alpha }\left(T\right)$ behave in a very similar manner for temperatures approaching $T_{g}$ and that the fragility of the two samples is thus similar. The fragility parameter (m) for the isotropic and nematic LCEs can also be determined from the VFT parameters, and the results are m= 110 and m= 130 for the isotropic and nematic LCE, respectively. This demonstrates that both LCE phases are fragile glass-formers with fragility values consistent with those of polymers [23,30]. For $T>T^{*}$, however, both the isotropic and nematic LCEs are less fragile which is demonstrated by the VFT parameters D=16.6 and D=17.2. The derivative analysis demonstrates that the nematic LCE can be well described as Arrhenius in this T-range. The gradient of the Stickel plot for $\;T>T^{*}$ is −0.04 for the nematic LCE which is very close to Arrhenius behavior (gradient of 0). Thus, an Arrhenius fit is also applied to this region of the data of the nematic LCE (Figure 3b, solid blue line). The result of the Arrhenius fit is $1.15\times 10^{-29}\;s$ and 151 ${\mathrm{kJ}\;\mathrm{mol}}^{-1}$. Interestingly, following the same trend towards more Arrhenius-like behavior above T*, the isotropic LCE is less non-Arrhenius above, than below, $T^{*}$.

Finally, we find that, $T^{*}$ is situated well above $T_{g}$ ($T^{*}/T_{g}\approx \;$ 1.17) and corresponds to $\tau_{\alpha }\left(T^{*}\right)$ = $3.8\times 10^{-5}\;s$ and $5.0\times 10^{-6}\;s$ for the isotropic and nematic LCE samples, respectively. Importantly, the ratio $T^{*}/T_{g}$ for the cross-over in behavior in the LCEs is close to the ratio $T_{B}/T_{g}$ observed for conventional glass formers ($T_{B}/T_{g}$ = 1.2–1.6) [30], suggesting a related origin. However, for conventional non-LC glass formers, $\tau_{\alpha }\left(T\right)$ typically transitions to more markedly non-Arrhenius (more fragile) behavior for $T>T_{B}$, whereas we observe the opposite trend for $T>T^{*}$. Moreover, for non-LC glass-formers, a bifurcation scenario resulting in a Johari–Goldstein β relaxation at $T_{\alpha \beta }\sim T_{B}$ is often observed. Neither of the two dielectrically active secondary relaxations, β or γ, observed in this work show any relation with $T^{*}$. We note, however, that we cannot rule out the existence of another secondary relaxation that is not dielectrically active (and thus not detected in our measurement), which demonstrates a bifurcation behavior near $\;T^{*}$.

In SCLCPs, a qualitatively similar $\tau_{\alpha }\left(T\right)$ behavior is observed for the α relaxation, where an Arrhenius behavior describes the data for $T>T^{*}$, and a VFT behavior for $T<T^{*}$ [42,46,57]. Temperature ratios of $T^{*}/T_{g}$ = 1.1–1.3 have been observed in nematic and smectic SCLCPs [42,46], which is close to the ratios observed for our LCE. In studies of homologous series of methacrylate-based SCLCPs with systematically varying side-chain length, the $T^{*}/T_{g}$ ratio was found to be fixed, independent of the phase transition temperatures, thus supporting the independence of the crossover behaviour on the LC phase behavior [42,46]. The activation energy, $\Delta E_{A}$, for the α relaxation of SCLCPs in the high-T Arrhenius regime typically ranges from 80–127 ${\mathrm{kJ}\;\mathrm{mol}}^{-1}$ [42] and can be compared to the value of 151 ${\mathrm{kJ}\;\mathrm{mol}}^{-1}$ obtained in our work. Thus, the behaviors observed for SCLCPs are generally very similar to the observations for our LCE. 

The cross-over behavior observed for SCLCPs has often been suggested to be related to a matching between the characteristic length-scale of correlated motions involved in the structural α relaxation, (often discussed in terms of a cooperatively rearranging region, or CRR) and a length-scale characterizing microphase separation of mesogen-rich and polymer-rich domains [22,42,46]. Microphase separation has been observed in polysiloxane SCLCPs that readily phase separate [58] and in SCLCPs which form layers due to smectic phase behavior. However, we observe cross-over behavior at $T^{*}$ in both the nematic LCE and isotropic LCE; microphase separation is certainly not present in our isotropic LCE. Thus, at least for our LCE, the origin of the observed crossover behavior lies elsewhere, and we will return to this in Section 2.3. 

### 2.2. Ionic Conductivity Behavior of the Isotropic and Nematic LCE

There is strong interest in developing polymer-based materials for applications in energy materials, such as batteries, e.g., as electrolytes or electrode binders [13,59]. Polymer-based electrolytes could, e.g., provide both the safety, mechanical flexibility and rigidity needed for ion-transporting membranes to act simultaneously both as ion conductors and electrode separators. Elastomers, in particular, show promise since their cross-links impart mechanical stability while $T_{g}$ can be kept relatively low, which provides mobility and thus more efficient ion transport. However, polymer-based materials still have relatively high $T_{g}$ values, which means that if ion transport is strongly coupled to the structural relaxation, sufficient ion transport is very difficult to achieve [60,61]. Thus, it is of significant interest for future applications to understand how to control the coupling of ion transport and structural relaxation. Moreover, polymer systems with LC functionalities have been identified as interesting candidates for battery applications [62,63] due to the additional structural control provided, which can affect both the nature and efficiency of the ion transport, as well as allow for anisotropic control of charge transfer. LCEs are particularly interesting in this respect due to their cross-linked nature which results in a combination of mechanical rigidity and liquid crystalline functionalities. However, there have been few studies to date exploring this for LCEs [64,65].

Studies of conventional polymeric materials have shown that for relatively low-$T_{g}$ polyethers, such as PEO and PPG, the structural α relaxation and the ionic DC-conductivity (σ) are highly coupled whereas higher $T_{g}$, less flexible polymers such as polycarbonate and poly(methyl methacrylate) show significant decoupling [60,66,67]. It is noteworthy that the well-coupled polyethers have strongly ion-coordinating ether oxygens spaced regularly along the backbone and ion transport has been shown to preferentially take place along the chain for these [67,68]; thus, these commonly used ion-conducting polymers might be regarded as outliers compared to polymers without such clear coordination structures. The detailed origin of the ‘decoupling behavior’ is however not presently well understood. For inorganic superionic glasses which demonstrate very strong decoupling, models exist which are typically focused on understanding the contributions to the fixed energy barrier that controls ion motion in the glass, e.g., from electrostatic and elastic forces [61]. Similar approaches could be adapted also for polymeric materials, where T-dependent changes in elastic and dielectric properties for $T>T_{g}$ would result in a T-dependent barrier, as typically described using a VFT expression [61], and a corresponding degree of decoupling.

The decoupling parameter, γ, is a way to quantify the extent of the decoupling of the ionic conductivity from the α relaxation and is determined from the relationship $\sigma \propto \tau_{\alpha }^{-\gamma }$. For long-chain polymers, a variation of the decoupling parameter between different polymers have been observed, and a rough trend was suggested where polymers with higher fragility [60] (typically also higher $T_{g}$) showed more decoupling than less fragile polymers. Dynamic fragility can typically be related to molecular packing and as a rule of thumb it was thus proposed that more fragile polymers pack less effectively, and thus leave more space for ions to move without assistance from matrix relaxations [60,66]. 

**Figure 4:**
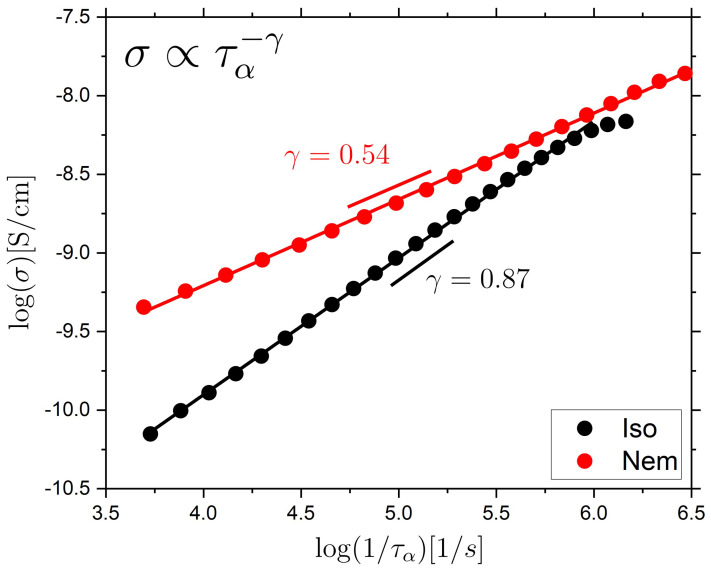
Plot of $\log\left(\sigma \right)\;\mathrm{vs}.\;\log\left(1/\tau_{\alpha }\right)$ for the isotropic and nematic LCE samples. Furthermore shown is the γ coupling coefficient from the equation $\sigma \propto \tau_{\alpha }^{-\gamma }$, which is related to the extent of coupling between the α relaxation and the ionic conductivity.

In our study, we have not specifically added ions. However, our LCE samples contain a small number of ionic impurities and we determined the corresponding ionic DC-conductivity and investigated how this correlates with the structural relaxation, and importantly identify the effects of the LC phase on this behavior. To determine the decoupling parameter, γ, we plot the measured ionic conductivity vs. the inverse structural α relaxation time for both the isotropic and nematic LCEs in a double-logarithmic representation, as shown in Figure 4. This type of Walden-like plot [59] is often used to investigate the relationship between ionic DC-conductivity and structural relaxation, and a slope near 1 indicates a strong coupling, whereas a smaller γ indicates decoupling. We find that the isotropic LCE is relatively well coupled with a coupling parameter of γ = 0.87, whereas the nematic LCE is significantly less coupled, corresponding to a γ = 0.54. The fragility of the isotropic and nematic LCE is m= 110 and m= 130, respectively. These two fragility values are quite similar, but we still note that a higher fragility is typically associated with a stronger decoupling in conventional polymeric materials [60].

For non-polymeric glass-formers, the Stokes–Einstein (SE) relation relates the translational diffusion coefficient D to the viscosity η, according to $D=k_{B}T/a\eta r$, where *r* is the radius of the diffusing entity, a is a constant, and T is the temperature. The SE relation often holds both for probe diffusion and molecular self-diffusion for $T>T_{B}$; thus, $D\propto \eta^{-1}$. Moreover, since $\eta \sim \tau_{\alpha }$ [69] to a good approximation, $D\propto \tau_{\alpha }{}^{-1}$. However, for $T<T_{B}$, a more complex ‘fractional’ SE behavior is often observed instead, where D∝ $\tau_{\alpha }{}^{-\zeta }$, with ζ~0.6−0.9 [70,71]; ζ has been reported to vary with fragility [72]. Since the ionic DC-conductivity is proportional to the diffusion coefficients for the ions in the material, the similarity between these observations and the observations for ion-conducting glass-forming materials, described above, is evident. 

A commonly invoked explanation for the ‘breakdown’ of the SE-relation is that it is caused by the development of dynamic heterogeneities for $T<T_{B}$, i.e., different spatial regions in the liquid are characterized by different characteristic relaxation times. The detailed link is not presently clear, but it has been argued that the SE-breakdown occurs since D and η (or $\tau_{\alpha }$) are averaged differently over the heterogeneous distribution of environments [73,74]. Alternatively, it has been suggested that  D and η (or $\tau_{\alpha }$) couple differently to spatial variations in intermolecular cooperativity or that the relationship is affected by the presence of an emerging secondary relaxation mechanism [75]. In summary, the detailed picture is presently not clear and more work is clearly needed to determine these links. 

For LC-based materials, a number of studies have investigated the coupling between ion conduction and structural relaxation [15,17,76]. In 5CB, in the isotropic phase on the approach of $T_{NI}$, a strong deviation from a Walden-plot gradient of 1 was observed, indicating significant decoupling, which was explained as due to the presence of pre-transitional nematic fluctuations [16]. The addition of nanoparticles to the isotropic phase of 5CB was shown to reduce the decoupling between DC-conductivity and the α relaxation and the behavior was attributed to the nanoparticle-induced disruption of pre-transitional nematic fluctuations in the isotropic phase [76]. The nematic phase shows a distribution of nematic domains with slightly varying order parameters and orientations throughout the sample, and is therefore likely to be more dynamically heterogeneous than the isotropic phase, which contains only pre-transitional nematic effects. Thus, explanations focused on the presence and strength of dynamic heterogeneities, and how these influence both the structural relaxation and ionic conductivity, might explain the observations of a greater decoupling in the nematic than in the isotropic phase observed in our LCE system and LC systems in general. 

Finally, it is important to note that for the nematic LCE, the mesogenic units are arranged in an ordered manner which could in itself affect the transport of ions. Effects on ionic transport, and the relationship between the DC-conductivity and the structural relaxation, due to induced spatial anisotropies have also been observed for the non-LC polymer PEO, where the PEO chains were aligned either by mechanical stretching or by magnetic and electric fields [68,77]. The observed effects were interpreted as due to molecular structure-induced changes in conduction pathways. For LC-based systems, the structural organization characterizing some phases, could thus directly affect both the efficiency of the ion transport, as well as the coupling between the ion transport and the structural relaxation. What is clear from our study is that LCEs containing relevant ion-coordinating chemistries should be highly interesting materials for which the LC phases can be utilized to tune the ion transport properties.

### 2.3. Rheology of the Isotropic LCE Sample

**Figure 5:**
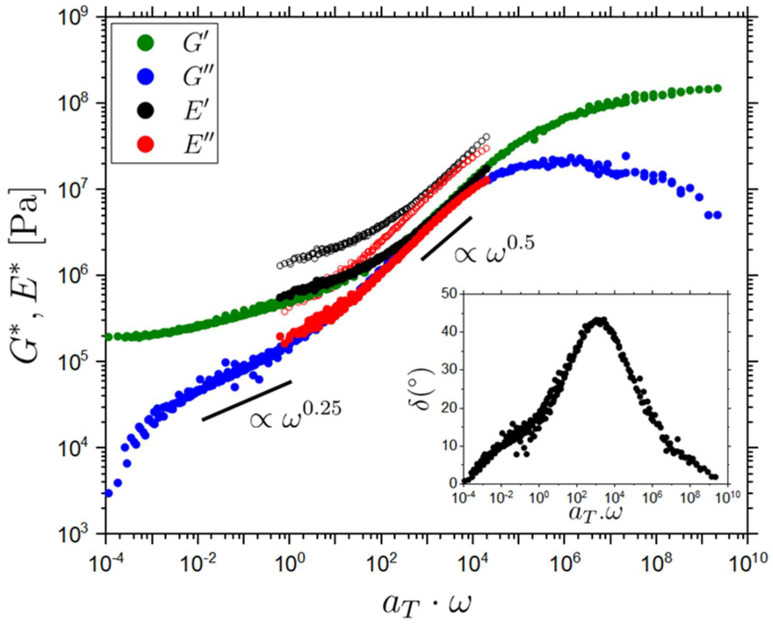
TTS master curves for shear storage modulus ($G^{\prime }$, green circles), shear loss modulus ($G^{\prime \prime }$, blue circles), unshifted storage modulus ($E^{\prime }$, hollow black circles), unshifted loss modulus ($E^{\prime \prime }$, hollow red circles), shifted storage modulus ($E^{\prime }$, black circles) and shifted loss modulus ($E^{\prime \prime }$, red circles). A vertical shift of −0.37 (on the log-scale) is applied between $G^{*}$ and $E^{*}$ demonstrating a very good agreement between the two data sets. Approximate power-law scalings of $\omega^{0.5}$ and $\omega^{0.25}$ discussed in the text are illustrated. Inset: phase angle ($\delta =\tan^{-1}\left(G^{\prime \prime }/G^{\prime }\right)$) against TTS shifted angular frequency ($a_{T}.\omega$) for the SAOS data.

The rheological behavior of LCEs is fundamentally interesting due to the coupling of the mesogenic units to the polymeric network. Whilst there have been previous studies on the dynamic rheological behavior of LCEs these have largely been performed on LCEs with polysiloxane backbones and/or LCEs which deform via the SSE response [48,49,50,78]. Here, we determined the rheological behavior of our isotropic LCE, which in contrast to these literature studies has an acrylate-based backbone. We studied the LCE using Dynamic Mechanical Analysis (DMA) and Small Amplitude Oscillatory Shear rheology (SAOS). To obtain data over a wide frequency range, we used Time Temperature Superposition (TTS) to construct master curves (see the Section 3 for a detailed description of the procedure). The validity of TTS was first investigated by plotting the data in a so-called van Gurp–Palmen representation (see Supplementary Materials) which removes all explicit time-dependence from the unshifted rheological data and therefore, shows if accurate TTS using frequency shifts is possible [79]. Both the DMA and SAOS data fall on a single line on the van Gurp–Palmen plot, respectively. Thus TTS can be adequately performed on these samples with *T* = 40 °C selected as the reference temperature. A horizontal shift factor is applied to the data at other temperatures to form a master curve (see Materials and Methods for details). The resulting rheological master curves are shown in Figure 5, SAOS is used to determine the complex shear modulus, $G^{*}\left(\omega \right)=G^{\prime }\left(\omega \right)+iG^{\prime \prime }\left(\omega \right)$, ($G^{\prime }$ = green circles, $G^{\prime \prime }$ = blue circles) and DMA is used to determine the complex Young’s modulus $E^{*}\left(\omega \right)=E^{\prime }\left(\omega \right)+iE^{\prime \prime }\left(\omega \right)$ ($E^{\prime }$ = hollow black circles, $E^{\prime \prime }$ = hollow red circles). The DMA data are shifted (shifted $E^{\prime }$ = black circles, shifted $E^{\prime \prime }$ = red circles) to directly compare the SAOS and DMA data to each other. As seen in Figure 5, $E^{\prime }$ and $E^{\prime \prime }$ can be collapsed onto $G^{\prime }$ and $G^{\prime \prime }$ (vertical shift of −0.37 applied). The Poisson ratio for the isotropic LCE can be determined from these data using the relationship:

(6) $$G^{\prime }=\frac{E^{\prime }}{2\left(1+\nu \right)}\;,$$

where $G^{\prime }$ and $E^{\prime }$ are the shear and elastic storage moduli respectively and ν is the Poisson’s ratio. The Poisson’s ratio is determined in the region where we have data for both $E^{\prime }$ and $G^{\prime }$, i.e., between ~0.6 and 1.9 × 10^2^ rad/s. The mean value of the Poisson ratio over the full range of the rheological data is 0.19 ± 0.03. The value of the Poisson’s ratio at 1 rad/s is 0.25 ± 0.05, where this data has been selected for the reference temperature and therefore removes any added complications resulting from TTS shifting. These determined values of the Poisson’s ratio (0.19 ± 0.03 and 0.25 ± 0.05 respectively) fall within the physical limits of the Poisson’s ratio for isotropic materials (−1≤ν≤0.5), and are comparable to those determined in previous studies for polydomain LCEs, where 0.2<ν<0.35 was observed for small strains, depending on the cross-link density [80,81].

The rheological data in Figure 5. includes the structural α-relaxation response in the high-frequency range ($\sim 10^{7}-10^{10}$ rad/s). At lower frequencies in the range of $\sim 10^{2}-10^{4}$ rad/s, a power law-like regime is observed where $G^{\prime }=G^{\prime \prime }\propto \omega^{0.5}$, and this scaling is more pronounced for $G^{\prime \prime }$ due to the transition towards a rubber-like plateau at low frequencies in $G^{\prime }$, resulting from the presence of permanent cross-links. The observed scaling is evidence of a Rouse-like spectrum [82,83]. A Rouse-like mode spectrum has similarly been reported in isotropic, nematic and smectic LCE systems [48,49,50,78]. Towards lower frequencies ($10^{1}-10^{-2}$ rad/s), a flatter approximate power-law-like regime of $G^{\prime \prime }\propto \omega^{0.25}$ is observed. This contribution is also observed as a shoulder in the low-frequency flank of the peak in the loss angle (δ) shown in Figure 5 (inset, $\tan\delta =G^{\prime \prime }/G^{\prime }$). Similarly, a low-frequency power law of $G^{\prime }\approx G^{\prime \prime }\propto \omega^{0.3}$ has been observed in the SmA phase of LCE systems, whereas it was not observed for the corresponding isotropic phase [50,78]. This scaling in SmA LCEs was interpreted as due to the presence of smectic layers which influence the otherwise separated polymer backbones [50,78,84]. However, for our isotropic LCE this situation is clearly not the same, and the observed behavior must have a different origin. Our LCE is a randomly cross-linked network containing the pendant units A6OCB and EHA. Based on. this, we propose two mechanisms for the low-frequency behavior. Firstly, the observed response could be related to the motion of free chains through the network. A $G^{\prime \prime }\propto \omega^{0.2-0.3}$ dependence has indeed been reported in cross-linked poly(dimethylsiloxane) (PDMS) networks where linear ‘free’ PDMS chains were present [85]. Secondly, the relaxation could be due to the motions of the dangling pendant chains within the network. This has been observed for poly(butyl acrylate) networks, where it has been interpreted as due to chain motions linked to pendant arm retraction, [86], and in PDMS networks with pendant chains where the details of the loss contribution depended on the pendant chain length [87].

**Figure 6:**
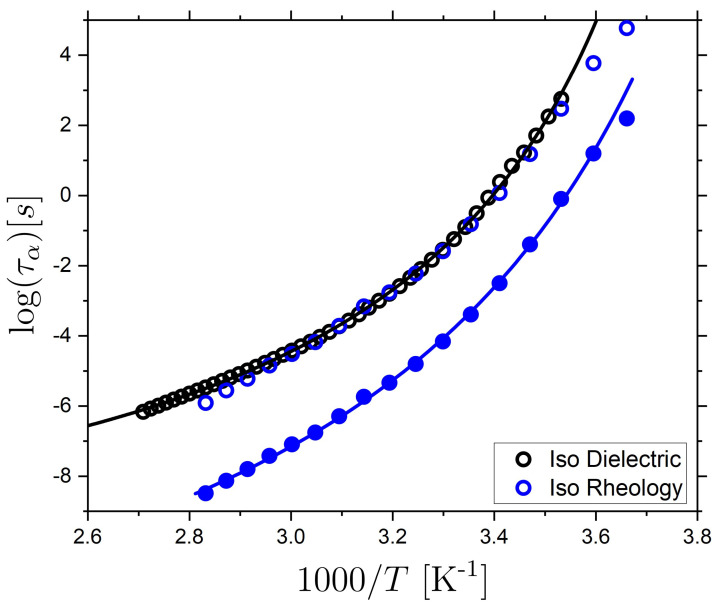
Arrhenius plot showing the α relaxation time, $\tau_{\alpha }\left(T\right)$ vs. inverse T for the isotropic LCE as measured using BDS (black hollow circles) and SAOS (filled blue circles). The SAOS data are also shown with a vertical shift of 2.58 (blue open circles), demonstrating the similarity in the T-dependence of the α relaxation, as probed by the two techniques. VFT fits of the BDS and SAOS data are shown in solid lines.

From the TTS shift-factors, we directly obtain information about the T-dependent characteristic time-scale for the LCE within the investigated T-range. The α relaxation timescale at the reference T (40 °C) is determined from the peak maximum in $G^{\prime \prime }$. Subsequently, $\tau_{\alpha }\left(T\right)$, from the rheological data, is determined by applying the $a_{T}\left(\omega \right)$ to $\tau_{\alpha }(T_{0}=40$ °C) (see Section 3 for details). The temperature dependence of the α relaxation determined from rheology is compared to our results for BDS in Figure 6. Here, a vertical shift of 2.58 (on the log scale) is applied to the rheology data set to overlay it with the BDS data. A shift of the relaxation time scales is expected between rheological and dielectric measurements [88] and based on this analysis, good agreement is found between the two data sets. Next, the rheology data are fit with a VFT expression and the results of the fits are shown in Table 3. Importantly, the close correspondence between the two data sets supports the validity of the TTS approach used in our analysis. 

### 2.4. Volume of Correlated Motions in the Isotropic LCE

As the glass transition is approached, the α relaxation is characterized by the presence of dynamic heterogeneities, i.e., regions in space with dynamics different from their surroundings. The size of the spatial regions of correlated motion has been determined for both non-polymeric and polymeric liquids [89], using a range of techniques, including NMR [90], modulated DSC [91], dielectric spectroscopy [89], or MD-simulations [92]. The determined length-scale characterizing correlated motions is typically ~1–5 nm [91,93] in the vicinity of $T_{g}$, where the detailed behavior depends on the specifics of the system, such as its dynamic fragility. Here, we use our BDS data to estimate the characteristic length scale (see the Section 3 for a detailed description). The dynamic heterogeneity is linked to fluctuations of the dynamics in time and space and these can be characterized by a so-called 4-point dynamic susceptibility $\chi_{4}\left(t\right)$ that quantifies the amplitude of spontaneous fluctuations around the average dynamics [94]. $\chi_{4}\left(t\right)$ can be expressed as a correlation, in time and space, of 2-point correlators that are readily experimentally accessible, e.g., through BDS. $\chi_{4}\left(t\right)$ is typically a non-monotonic function with a peak occurring near the α-relaxation time, and a height that is proportional to the volume of correlated motions, $V_{\mathrm{corr},\;4}$, or alternatively the number of molecular units that undergo correlated motions within this volume, $N_{\mathrm{corr},4}$ [94]. It is difficult to experimentally directly measure spontaneous fluctuations and thus $\chi_{4}\left(t\right)$, however, using a fluctuation-dissipation relation, it has been demonstrated that one can approximate $\chi_{4}\left(t\right)$ by determining induced fluctuations, e.g., how the relevant 2-point correlator (experimentally readily available) responds to a perturbation, such as temperature [89,94]. 

**Figure 7:**
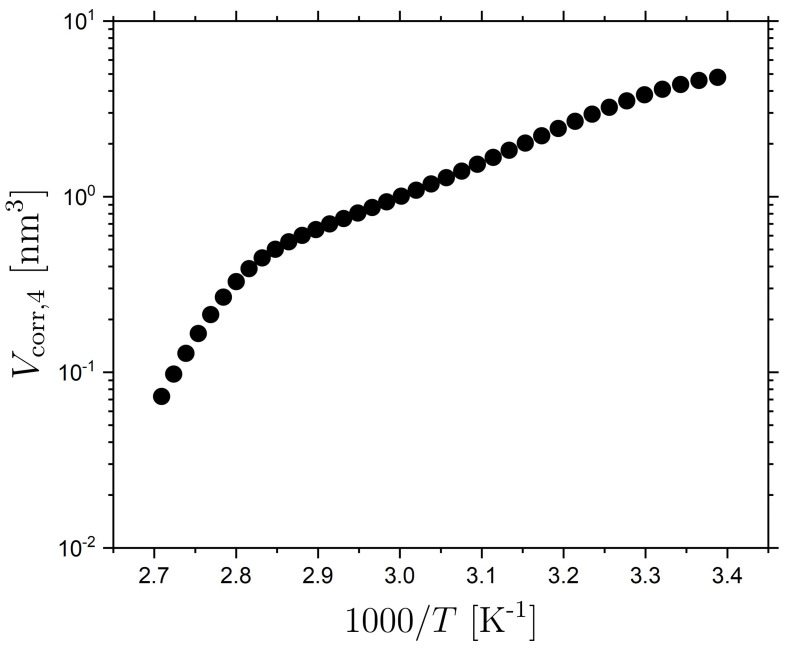
Volume of correlated molecular motions for the isotropic LCE sample as a function of inverse temperature, as discussed in detail in the text.

Here, we use this technique, as outlined in detail in the Section 3, to determine an estimate of $V_{\mathrm{corr},4}$. The T-dependent correlation volume $V_{\mathrm{corr},4}$ estimated from the approach outlined above is shown in Figure 7 as a function of inverse temperature for the isotropic LCE; in the Supplementary Materials $V_{\mathrm{corr},4}$ is also plotted versus $\tau_{\alpha }$. The observed behaviour is typical for glass-formers with an increasing $V_{\mathrm{corr},4}$ for decreasing T and the stronger T-dependence at higher temperatures, which significantly reduces near $T_{g}$ [89,95]. We do note that the most significant change in T-dependence of $V_{\mathrm{corr},4}$ takes place at around 350 K (1000/T∼ 2.86), which is above the range where we observe changes in $\tau_{\alpha }\left(T\right)$ at $T^{*}=$333 K. Importantly, we find an estimate for the volume of correlated motions for the isotropic LCE at the transition temperature $T^{*}$ of ~1 nm^3^, corresponding to a length-scale of $l_{\alpha }\left(T^{*}\right)∼$1 nm.

Given that $\tau_{\alpha }\left(T\right)$ undergoes a crossover to more Arrhenius-like behavior for $T>T^{*}$ for both the nematic and isotropic LCE, which is opposite to the crossover to more non-Arrhenius behavior for non-LC glass-formers, it is interesting to compare $l_{\alpha }\left(T^{*}\right)$ to any length-scale related to nematic behavior for the ‘isotropic’ LCE. The most pertinent length-scale is the static correlation-length ξ of pre-transitional nematic domains [96]. The correlation length of the pre-transitional nematic regions (ξ) in an isotropic phase follow the equation [96]:

(7) $$\xi =\xi_{0}\sqrt{\frac{T_{c}}{T-T_{c}}},$$

where $\xi_{0}$ is the bare correlation length of the pre-transitional nematic regions with $\xi_{0}∼0.5\;\mathrm{nm}$ in simple molecular LCs [96], $T_{c}$ is a supercritical temperature which is typically ∼1 K lower than $T_{NI}$. The isotropic phase is templated into the LCE and after polymerization there is no evidence of a phase change in the isotropic LCE as investigated by DSC [4]. We expect that the pre-transitional nematic regions are frozen-in during polymerization, a phenomenon similar to ‘frozen-in order’ near cross-linking points and ‘quenched disorder’ previously observed in LCEs [97,98]. Our isotropic LCE is polymerized at 60 °C and the $T_{NI}$ of the precursor mixture is 36 °C [4,20]; therefore by substituting these values into the above equation, we expect that the correlation length will be of the order of 3.5$\;\xi_{0}$. Using the typical value of $\xi_{0}=$ 0.5 nm, we thus expect the correlation length of the nematic domains in the isotropic LCE to be of the order of ξ = 1.8 nm. This is comparable to the length scale of correlated motions in the α relaxation at $T^{*}$. Thus, it seems plausible that the difference in $\tau_{\alpha }\left(T\right)$ in the isotropic and nematic phase is related to the interplay between length-scales characterizing the correlated molecular (segmental) motions of the α relaxation and the correlation length of the pre-transitional phenomena in the form of nematic fluctuations of the isotropic LCE. 

The results of this analysis are in general agreement with studies of the structural relaxation in molecular LCs [15,16,17,99]. It has been shown that the temperature-dependent behavior of the structural relaxation of LC materials is strongly influenced by pre-transitional phenomena in the isotropic phase [15]. It has also been shown that the addition of nanoparticles disrupts the pre-transitional phenomena present in the isotropic phase [76,100]. With low concentrations of nanoparticles, a cross-over from non-Arrhenius to Arrhenius behavior has been observed in the isotropic phase; further increase in the nanoparticle concentration causes a cross-over from Arrhenius to non-Arrhenius behavior [100]. Hence the difference in $\tau_{\alpha }\left(T\right)$ in the isotropic and nematic LCE could be understood by the presence of pre-transitional nematic regions and nematic correlations, respectively, and the relative size of these with respect to the length-scale of correlated motions of the α relaxation. Previous measurements on SCLCPs in their isotropic phase, thus lacking pre-transitional nematic fluctuations, have shown that $\tau_{\alpha }\left(T\right)$ can be described with a single VFT [42], as is also typical for polymeric materials with a sufficiently long chain-length [24]. In contrast, the precursor mixture presented herein has a nematic to isotropic transition and thus, in the isotropic phase, will have pre-transitional nematic regions present.

### 2.5. Effect of Strain on the Dielectric and Rheological Behaviors of the Nematic Sample

In previous work on our LCE system [18,19], it has been demonstrated that the nematic phase shows a complex response to an imposed uniaxial deformation; this includes linear and non-linear elastic behavior, a reduction in uniaxial order, the emergence of biaxial order, and molecular auxetic response [18,19]. The stress–strain behavior has been reported previously to reveal an initial linear elastic regime, followed by a plateau-like softening behavior, and a subsequent growth of the stress [14,20]. The plateau and subsequent stiffening of the stress–strain curve have been observed in both the engineering stress–engineering strain and true stress–engineering strain and are therefore not a consequence of sample necking [14]. A softening of the stress–strain response is a hallmark of the ‘semi-soft elastic response’ [5,47], which is due to the continuous rotation of the nematic director in counter-rotating domains known as stripe domains. However, it has been previously demonstrated that the behavior observed for our nematic LCE is instead due to a mechanism known as the mechanical Frèedericksz transition, which is signified by a discontinuous rotation of the nematic director [19,20]. Investigations into the order parameter of our nematic LCE have shown a link between the mechanical Frèedericksz transition, reduction in uniaxial order and the emergence of biaxial order with imposed strains [19]. To understand these observations, it is important to also identify, and understand, the effects of deformation on the rheology and relaxation dynamics of the nematic LCE. To achieve the former, the complex Young’s modulus was determined using a Dynamic Mechanical Analyzer (DMA) (see Section 3 for details). Measurements were performed on a sample with dimensions 5 cm × 0.2 cm × 100 µm. The LCE film was subjected to varying elongations, and thus strains, ranging from 0 to 120%. The elongations of the LCE sample is shown in true strain $(\epsilon_{t}$) representation:

(8) $$\epsilon_{t}=\ln\left(\frac{L_{f}}{L_{i}}\right),$$

where $L_{f}$ is the length of the sample after elongation, and $L_{i}$ is the initial sample length. After the applied elongation, the LCE is left to stress–relax for 2 min, where this time is selected to be sufficiently short to avoid sample breakage, yet long enough to not affect the auxetic response of the material [18]. Similar relaxation times have been used to investigate the tensile mechanical response of the material and the order parameter behavior [14,19]. For each elongation, its relevant storage and loss elastic modulus ($E^{\prime }\left(f_{0}\right)$ and $E^{\prime \prime }\left(f_{0}\right)$) determined for $f_{0}=1$ Hz (*ω* ≈ 6.3 rad/s) were determined by applying oscillatory strains of 0.1% to the pre-elongated sample; oscillatory strains of 0.1% were confirmed to be in the linear viscoelastic region of the nematic LCE via a strain sweep. For comparison, tensile stress–strain measurements, published previously [20], are shown in the inset from which the Young’s modulus (E) was determined. The results and methodology of the tensile measurements have been reported in full elsewhere [18], but briefly, tensile stress–strain measurements were performed in a bespoke rig consisting of two actuators and a load cell enclosed in a temperature-controlled environment; images of the LCE are recorded with a camera and changes in length and width of the sample are determined which allows for the calculation of the true-stress and true-strain of the LCE. The trues tress is defined as $\sigma_{t}=F/A$, where F  is the force measured on a sample and  A is the cross-sectional area of the sample after the application of strain. The cross-sectional area of the LCE as a function of strain is calculated using the assumption of constant volume, which has previously been shown to be a good approximation for this LCE [18].

The effects of elongational strain on the mechanical response (e.g., the Young’s or shear moduli) is an important consideration in understanding materials under strain [101,102]. Changes in loss moduli could, e.g., be related to the breaking of bonds or molecular slippage occurring within polymers [101]. Other examples include experiments on natural rubber and styrene butadiene rubber for which strains < 170% did not affect the complex moduli (for f=1 Hz), whereas at larger strains an increase was observed in both storage and loss moduli [103] which was assigned to an increase in the effective constraints on the molecular orientations within the elastomer network due to the applied strain [104], and to the finite extensibility of the network [105]. 

**Figure 8:**
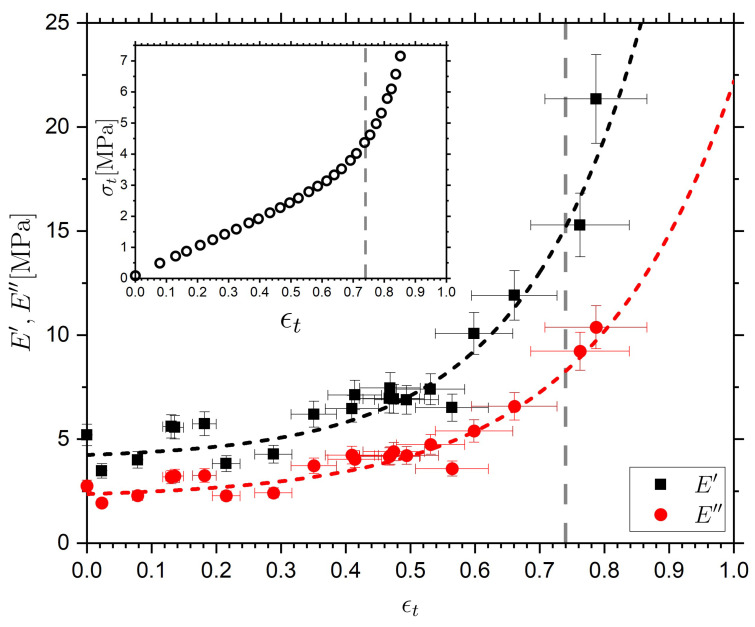
Storage and loss moduli determined at 1 Hz (2π rad/s) and T = 21 °C, as a function of true strain applied perpendicular to the nematic director. Inset: non-dynamic tensile tests showing the true stress (hollow circles) ($\sigma_{t\;}\left[\mathrm{MPa}\right])$ as function of true strain $\epsilon_{t}.$ The grey dashed line marks the threshold for the onset of the molecular auxetic response.

Figure 8 shows the evolution of $E^{\prime }$ and $E^{\prime \prime }$ for the nematic LCE as a function of external strain applied perpendicular to the nematic director. The grey dashed line in Figure 8 denotes the onset of the molecular auxetic response that has been reported for this system; this response is related to out-of-plane rotations of the mesogenic units [18,19]. The black and red dashed lines are exponential growth functions which serve as guides to the eye. At low values of applied strain ($\epsilon_{t}<0.22$), $E^{\prime }(f_{0}$) and $E^{\prime \prime }(f_{0}$) are relatively constant with average values of 4.9  ±  0.2 MPa and 2.7  ±  0.1 MPa respectively; the elastic modulus determined from the gradient of the true stress–true strain data in this region ($\epsilon_{t}<0.22$) is 4.6 MPa showing an excellent agreement with our determined $E^{\prime }\left(f_{0}\right)$. Between $\epsilon_{t}=0.22$ and $\epsilon_{t}=0.53$ average values of $E^{\prime }$ = 7.7  ±  0.6 MPa and $E^{\prime \prime }$ = 4.7  ±  0.4 MPa are observed as compared to 4.9  ±  0.2 MPa and 2.7  ±  0.1 MPa for $\epsilon_{t}<0.22$; therefore in this regime ($\epsilon_{t}<0.55$) there is a small dependence of dynamic moduli on strain. Within error, $\tan\left(\delta \right)=E^{\prime \prime }/E^{\prime }$ is constant (tan(δ)= 0.55  ±  0.03 and tan(δ)= 0.60  ±  0.07 for $\epsilon_{t}<0.22$ and $0.22<\epsilon_{t}<0.55$ respectively) which suggest that the LCE is deforming elastically (albeit non-linearly) throughout this strain region. 

At larger values of strain ($\epsilon_{t}>0.55$) a stiffening of the stress–strain response is observed in the true stress–true strain representation, additionally a significant increase in $E^{\prime }\left(f_{0}\right)$ and $E^{\prime \prime }\left(f_{0}\right)$ is observed. At the strain corresponding to sample breakage ($\epsilon_{t}\approx 0.78$), $E^{\prime }\left(f_{0}\right)\;$ = 21.3 MPa which is consistent with the value of the elastic modulus E~24 MPa determined from the gradient of the true stress–true strain data in this region. All the way up to sample breakage, tan(δ) is constant, within error, and the sample thus behaves elastically throughout the whole operational strain range. We note that the upturn in the true stress–true strain response, and the corresponding increases in $E^{\prime }\left(f_{0}\right)$ and $E^{\prime \prime }(f_{0}$) occurs just before the sharp rotation of the director at the mechanical Fréedericksz transition, and the emergence of molecular auxetic response (signified by the grey vertical line) [18]. To summarize, the DMA measurements reveal that the LCE sample deforms elastically throughout the whole strain range, and the observed upturns in $E^{\prime }\left(f_{0}\right)$ and $E^{\prime \prime }\left(f_{0}\right)$ occur near, but before, the onset of the mechanical Fréedericksz transition. 

To further understand the effects of applied strain on the nematic LCE, the α relaxation response for different applied strains was investigated using BDS. Literature reports of BDS experiments on a lightly cross-linked (6.5% mol/m^3^) polyisoprene-based non-LC elastomer found no effects of an applied “static” strain on the α-relaxation [9]. However, a polyurethane-based elastomer containing 32.5 wt.% hard segments (4,4′-diphenylmethane diisocyanate and 1,4-butanediol) subjected to applied strains ranging from 0% to 300%, showed a broadening and slowing-down of the α relaxation, and an increase in the fragility parameter, m [9]. A similar increase in the fragility, and slowing down of the α relaxation has also been reported for a polyurea-based elastomer [106]. In both cases, the change in the dynamics of the strained elastomers was related to increasing constraints imposed on the soft segments of the elastomers due to a deformation-induced reduction in microphase separation [9,106]. Experiments have also been performed aimed at investigating the effects of mechanical deformations on the relaxation dynamics of polymer glasses [10,11,12,107]. Uniaxial deformation experiments performed on poly(methyl methacrylate) (PMMA) under either constant load or constant strain-rate demonstrate that below the yield stress, the mobility of the α relaxation is enhanced by stress which can be interpreted as due to tilting of the potential energy landscape, leading to a lowering of activation barriers [108]. Above yielding, more dramatic behavior can be observed with a strong sensitivity to applied stress or strain-rate, and significant effects on the observed spatial dynamic heterogeneity [12,107].

**Figure 9:**
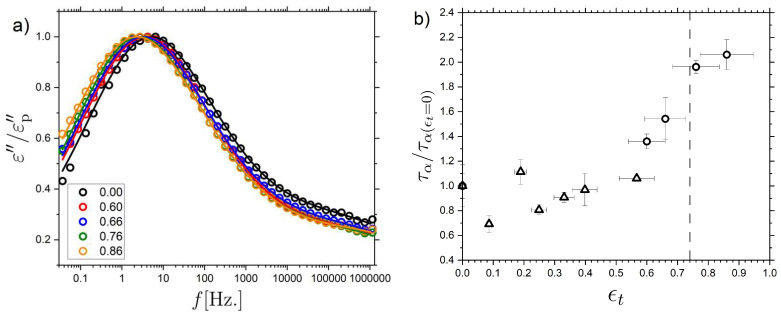
(**a**) Normalised dielectric loss $\varepsilon^{\prime \prime }$ versus frequency at *T* = 23 °C for nematic LCE sample, measured for a range of different applied true strains $\epsilon_{t}$, from 0.00 to 0.86 as shown in the legend. The solid lines are fit to the data, described in detail in the text. (**b**) The α-relaxation times $\tau_{\alpha }$ versus applied true strain, as determined from fits to the data shown in panel (**a**), and described in detail in the text. The grey dashed line marks the threshold for the onset of the molecular auxetic response.

To perform BDS measurements under strain, the nematic LCE sample was stretched to the desired strain and affixed to a 20 mm brass plate with Kapton tape. The sample dimensions were nominally 7 cm × 1.5 cm × 100 µm (L × W × T) and a brass plate of 5 mm was placed on top of the sample to allow for BDS measurements. It was confirmed that the Kapton tape was placed sufficiently far away from the electrodes to not influence the measurements. After mounting, the sample was left to stress–relax for 2 min before a measurement was performed. All measurements were performed at T = 23 °C to be able to directly compare with the DMA measurements described above. Figure 9 shows the frequency-dependent dielectric loss $\varepsilon^{\prime \prime }\left(f\right)$ data, as normalized by the maximum of the relaxation peak corresponding to the α relaxation, $\varepsilon_{p}^{\prime \prime }$. Data are shown for a set of applied strains varying over the range 0 to 140% ($\epsilon_{t}=0$ to 0.88). Within the measured dynamic range ($10^{-2}<f<10^{6}\;\mathrm{Hz}$), the α relaxation is observed together with the low-frequency side of the β relaxation. The data are thus fit using a sum of an HN-contribution (α relaxation) and a CC-function (β relaxation). We here focus on the α relaxation results, since the β relaxation contribution is only partly covered in the dynamic window. It is clear from Figure 9a that the general shape of the α relaxation remains the same even at large values of applied strain. The characteristic α relaxation times $\tau_{\alpha }$ (corresponding to the maxima of the loss peaks) obtained for increasing values of applied strain are shown in Figure 9b. Two separate LCE samples were investigated to determine the effect of strain on the α relaxation, one taken to smaller strain values ($\epsilon_{t}\leq 0.44$) (open triangles), and one taken to larger strain values ($\epsilon_{t}\leq 0.86$) (open circles) which is above the characteristic strain for the mechanical Fréedericksz transition where the LCE displays a molecular auxetic response ($\epsilon_{t}=0.73$). 

Due to slight differences in the unstrained α relaxation timescale between the two samples, the $\tau_{\alpha \left(\epsilon_{t}\right)}$ data are normalized by the unstrained timescale ($\tau_{\alpha \left(\epsilon_{t}=0\right)}$), the result of which is shown in Figure 9b. The dielectric spectra and unnormalized relaxation timescales are shown in the Supplementary Materials. For the sample subjected only to lower values of strain (Figure 9b, open triangles), we find no obvious trend in the $\tau_{\alpha }$ timescales within the accuracy of the data. However, in the sample taken to higher true strains (Figure 9b, open circles) there is a clear shift in the α relaxation to slower relaxation times. The results are therefore consistent with findings of a stress-relaxed polyurethane elastomer containing rigid units [9], which were attributed to increased strain-induced constraints on the polymeric backbone.

To summarize, both $E^{\prime }\left(f_{0}\right)\;$ and $E^{\prime \prime }\left(f_{0}\right)\;$ remain relatively unchanged with increasing strain until a large strain (~0.55) is imposed, after which an increase in both moduli is observed. Correspondingly, $\tau_{\alpha }$ remains relatively unchanged until an applied strain of ~0.55 after which the relaxation slows down. Thus, the changes in $\tau_{\alpha }$ and the complex modulus are observed for comparable values of true strain. (Figure 9b) Both of these effects could be understood in terms of a strain-induced increase of constraints, e.g., reflected in a reduced configurational entropy of the polymer backbone [9,109], and the finite extensibility of the network [105]. The increases in $E^{\prime }$, $E^{\prime \prime }$ and $\tau_{\alpha }$ occur near the discontinuous rotation of the director (the mechanical Frèedericksz transition) and the onset of the molecular auxetic response [18,19,20]. We cannot exclude the possibility that the observed behavior is a coincidence. However, we recently suggested that the auxetic response in our LCE is related to the emergence of biaxial order linked to out-of-plane rotations of the mesogenic units [19]. We propose that the out-out-plane rotations occur due to the strain-imposed configurational restrictions on the polymer backbone. 

## 3. Materials and Methods

### 3.1. Synthesis of the Liquid Crystalline Elastomer

**Figure 10:**
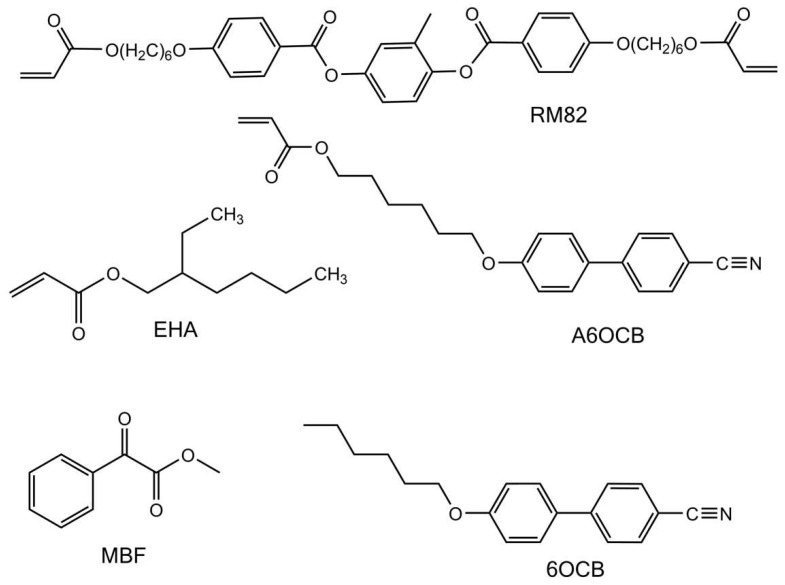
Structures of the constituent chemicals for both the nematic and isotropic LCEs.

The liquid crystalline elastomer used in this study was synthesized following a protocol published previously [4,18,20], and we thus only briefly describe the synthesis here. A mixture of the reactive mesogens 6-(4-Cyano-biphenyl-4′-yloxy)hexyl acrylate (A6OCB), 1,4-bis-[4-(6-acryloyloxyhex-yloxy)benzoyloxy]-2-methylbenzene (RM82) and the non-reactive mesogen 4′-Hexyloxybiphenyl (6OCB) is melted at 100 °C and subsequently cooled to 40 °C, and the non-mesogenic spacer 2-ethylhexyl acrylate (EHA) and photopolymeriser methyl benzoylformate (MBF) are added to form an isotropic mixture of the precursor chemicals. The molar ratios of this mixture are outlined in Table 4 and the corresponding chemical structures of the components are shown in Figure 10. The isotropic monomer mixture is capillary-filled at 40 °C into a cell with a spin-coated poly vinyl alcohol (MW = > 89,000, 0.5 %wt) layer that is rubbed to provide alignment. To form a monodomain nematic sample the sample is cooled to room temperature, into the nematic phase, and allowed to align for 20 min. The sample is polymerized using a UV curer (2.5 mW cm^−^^2^) for 2 h. An isotropic sample is prepared by capillary filling into an unaligned cell and curing at 60 °C. Both samples are washed with a 70/30% methanol/dichloromethane mixture to remove the unpolymerized 6OCB, and finally dried for 4 h at 60 °C. The prepared samples have nominal dimensions of 7 cm × 1.5 cm × 100 µm. 

### 3.2. Broadband Dielectric Spectroscopy 

Broadband dielectric spectroscopy was performed over a frequency range of $∼10^{-2}<f<10^{6}\;\mathrm{Hz}$. Using a Novocontrol Alpha-A dielectric analyser The LCE samples were sandwiched between two 10 mm diameter circular metal electrodes, separated by 100 µm using silica spacers. The temperature was controlled using a Novocontrol Quatro Cryosystem with an accuracy of 0.1 K and experiments were performed between −150 °C and 100 °C. The temperature-dependent complex permittivity was analyzed using a sum of relaxation contributions and a contribution from DC-conductivity. Each relaxation contribution was described using a Havriliak–Negami (HN) or Cole–Cole (CC) expressions [22,110], and the full relaxation spectrum is thus described by:

(9) $$\varepsilon^{*}\left(f\right)=\varepsilon_{\infty }+\overset{N}{\underset{j}{\sum }}\frac{\Delta \varepsilon_{j}}{{\left(1+{\left(i2\pi f\tau_{HN,j}\right)}^{p,j}\right)}^{q,j}}+\frac{-i\sigma }{2\varepsilon_{0}\pi f},$$

where $\varepsilon^{*}\left(\omega \right)$ is the complex permittivity, $\varepsilon_{\infty }$ is the high-frequency limit of the complex permittivity,  N is the total number of relaxation processes, and the index j refers to a particular relaxation. $\Delta \varepsilon_{j}$ is the dielectric strength, and $\tau_{HN,j}$ is a characteristic time-scale of relaxation j, and p,j and q,j are parameters characterizing the stretching of the *j*th relaxation process, respectively. σ is the DC-conductivity due to the presence of ionic impurities. The α relaxation was fitted using the HN function (p≠1, q≠1) whilst the β and γ relaxations are fitted using the CC expression (p≠1,q=1). We consistently use the maximum of the dielectric loss, corresponding to the most probably relaxation time, as the relaxation time characterising a particular relaxation. To obtain the time-scale corresponding to the peak maximum, $\tau_{p}\;$, we use the expression [22]:

(10) $$\frac{1}{\tau_{p}}=\frac{1}{\tau_{HN}}\left(\sin{\left(\frac{p\pi }{2+2q}\right)}^{1/p}\sin{\left(\frac{pq\pi }{2+2q}\right)}^{-1/p}\right).$$

### 3.3. Rheology 

Small amplitude frequency-dependent rheology was performed both in tension and shear. A Dynamic Mechanical Analyzer (DMA) (Rheometrics Solid Analyser; RSAII) with a film tension attachment was used to determine the frequency-dependent complex Young’s modulus $E^{*}\left(\omega \right)=E^{\prime }\left(\omega \right)+iE^{\prime \prime }\left(\omega \right)$, where $E^{\prime }$ is the storage and $E^{\prime \prime }$ the loss modulus. A strain of 0.1% ensured operation within the linear viscoelastic (LVE) regime, as confirmed by strain sweeps prior to the frequency scans. Frequency scans were subsequently performed between 6.3 and 78.5 rad/s from *T* = 22 °C to 48 °C in 2 °C steps. Moreover, Small Angle Oscillatory Shear (SAOS) measurements were performed on a Rheometrics ARES strain-controlled rheometer, using a liquid nitrogen cooling system in combination with a forced-convection oven. Samples were loaded between 5 mm and diameter parallel plates, using a gap of 0.5–1.0 mm. A strain-sweep test was carried out to ensure that measurements were performed in the linear regime, and frequency-scans were performed between 0.1 and 100 rad/s, every 5 degrees over the temperature range of 80 to 0 °C to encompass the α relaxation response. Upon lowering the temperature, the gap was reduced to ensure that the sample remained in the correct shape, and the strain was reduced to ensure an optimum torque (stress) response. 

For data from both DMA and SAOS, Time Temperature Superposition (TTS) was used to form master curves. TTS is relevant to use when a material, to a good approximation, is controlled by a single characteristic time-scale. Such thermorheologically simple [111] behavior has been shown to be a good approximation for some LCE systems [1,78,84]. We initially investigate the validity of TTS by plotting $\tan\left(\delta \right)\;\mathrm{vs}.\;\left|G^{*}\right|$ (or $\left|E^{*}\right|$) in a so-called van Gurp Palmen (vGP) plot (see Supplementary Materials) [79]. The vGP representation removes all explicit time-dependence from the unshifted rheological data and therefore, shows if accurate TTS using frequency shifts is possible. TTS was carried out by frequency (horizontal) shifting the $G^{\prime }$ and $G^{\prime \prime }$, or $E^{\prime }$ and $E^{\prime \prime }$, data to form a single master curve, yielding a frequency shift factor at each measured temperature ($a_{T}\left(T\right))$. The horizontal shift factors $a_{T}\left(T\right)$ are thus given by:

(11) $$\alpha_{T}=\frac{\tau \left(T\right)}{\tau \left(T_{0}\right)}=\frac{\omega \left(T_{0}\right)}{\omega \left(T\right)},$$

where τ(T) is the timescale of the response measured at T, and $\tau \left(T_{0}\right)$ is the timescale of the response at a reference temperature, $T_{0}$. A reference temperature of $T_{0}$ = 40 °C was used for both the SAOS and DMA data. When performing TTS analysis, a vertical shift factor, $b_{T}(T$) is sometimes required, e.g., to account for density changes in the material. However, for our LCE data, it was not necessary to invoke a vertical shift factor to describe our data, which is supported by the vGP analysis. To determine $\tau_{\alpha }\left(T\right)$ from the SAOS data, the characteristic time-scale of the α relaxation at $T_{0}\;$ was determined from the peak in $G^{\prime \prime }$. The shift factors at all other temperatures were used to determine $\tau_{\alpha }\left(T\right)$. 

The effect of applied strain on $E^{\prime }$ and $E^{\prime \prime }$ on the nematic LCE was investigated using DMA. The sample was held under strain between the RSAII tension clamps and stress-relaxed for 2 min. A 1 Hz oscillatory strain with an amplitude of 0.1% was applied at 23 °C to the strained nematic LCE and $E^{*}$ was determined. The sample is strained further, allowed to stress relax, and dynamic measurements are performed again. This allowed one to investigate $E^{\prime }\;$ and $E^{\prime \prime }$ as a function of applied strain up until sample failure. To correct for changes in cross-sectional area due to the applied strain, an approximation of constant volume was applied; this assumption is known to be a good approximation for the nematic LCE [18].

### 3.4. Differential Scanning Calorimetry and Modulated Differential Scanning Calorimetry

Conventional Differential Scanning Calorimetry (DSC), as well as modulated Differential Scanning Calorimetry (m-DSC), was employed to investigate the glass transitions of the isotropic and nematic LCEs. A TA Instruments Q2000 was used to perform both DSC and m-DSC. Approximately 10 mg of LCE sample was cut into 5 mm circular pieces and stacked into hermetic DSC pans. Conventional DSC was performed using a heating/cooling rate of 10 K/min. Each sample was held at 100 °C for 5 min to remove any thermal history. DSC heating/cooling runs were performed for 3 cycles between −70 °C and 80 °C, and the glass transition temperatures ($T_{g}$) were defined from the inflection points of the heat flow on the 2nd cooling cycle. m-DSC was performed using a linear heating ramp with a superimposed sinusoidal heating/cooling profile. A temperature amplitude of 1.2 K and a modulation period of 60 s was used with an underlying heating rate of 0.83 K/min across the glass transition regions. $T_{g}$ values were defined from the inflection point of the reversing heat flow A modulation period of 60 s corresponds to an α relaxation time scale of $\tau_{\alpha }=60/2\pi$ = 9.56 s. 

### 3.5. Determining the Correlation Volume from Broadband Dielectric Relaxation Data

Using our BDS data for the isotropic LCE, we define a normalized dynamic susceptibility $\chi \left(f,T\right)=\left[\varepsilon^{\prime }\left(f,T\right)-\varepsilon_{\infty }\left(T\right)\right]/\Delta \varepsilon \left(T\right)$, where $\varepsilon^{\prime }\left(f,T\right),\;\varepsilon_{\infty }\left(T\right)$ and Δε(T) are determined from the HN α relaxation contribution to the experimental data. Using the approximation discussed in the main text of references [89,94], it has been demonstrated that the number of correlated molecular units (molecules for a liquid or, e.g., monomers for a polymer), $N_{\mathrm{corr},4}$ can be determined as:

(12) $$N_{\mathrm{corr},4}\left(T\right)\approx \frac{k_{B}}{\Delta C_{p}^{mol}}T^{2}{\left\{\frac{max}{f}\left|\partial \chi \left(f,T\right)/\partial T\right|\right\}}^{2},$$

where $k_{B}$ is the Bolzmann constant and $\Delta C_{p}^{mol}$ is the isobaric configurational heat capacity per chosen molecular unit, directly related to the α relaxation. More precisely, Equation (15) is a lower bound for $N_{\mathrm{corr},4}$ but has been demonstrated to constitute a good approximation for both polymeric and non-polymeric systems [89,95]. $\Delta C_{p}^{mol}$ can be converted to a specific heat: $\Delta c_{p}$ = $\Delta C_{p}^{mol}\cdot N_{A}/m_{0}$, where $N_{A}$ is the Avogadro constant and $m_{0}$ is the molar weight of the chosen molecular unit. Moreover, we can define a correlation volume as:

(13) $$V_{\mathrm{corr},\;4}=N_{\mathrm{corr},4}\cdot \frac{m_{0}}{\rho \cdot N_{A}},$$

where ρ is the volumetric mass density. Thus, in conclusion, we find that:

(14) $$V_{\mathrm{corr},4}\left(T\right)\approx \frac{k_{B}}{\Delta c_{p}\rho }T^{2}{\left\{\frac{max}{f}\left|\partial \chi \left(f,T\right)/\partial T\right|\right\}}^{2},$$

where $\Delta c_{p}$ is the isobaric configurational specific heat associated with the α relaxation. Here, we determine $\Delta c_{p}$ directly from the measured specific heat step at $T_{g}$, We thus ignore the weak T-dependence of $\Delta c_{p}$. We also note that to estimate the contribution to the heat capacity arising from configurational degrees of freedom, it is common to subtract the contribution from the corresponding crystal, where mainly vibrational degrees of freedom contribute. However, since this is not possible for our material, we use the glassy state as a reference where relatively few configurational rearrangements take place and the vibrational contribution is dominating. The mass density, ρ, of the LCE is determined by measuring the mass of an LCE sample using a Mettler Toledo ME weighing scale, and by measuring the corresponding dimensions of the LCE sample using a Mituoyo Quantamike IP65 digital micrometer and calculating the volume of the sample. The density of the isotropic sample is 1300 ± 200 kg/m^3^ at 20 °C and we ignore any weak temperature dependence in ρ. We note that the primary contribution to $V_{\mathrm{corr},4\;}\left(T\right)$ arise from variations in $\tau_{\alpha }\left(T\right)$, which justifies our approximation of T-independent $\Delta c_{p}$ and ρ. We determined ∂χ(f,T)/∂T using finite differences as:

(15) $$\frac{\partial \chi \left(f,T\right)}{\partial T}\approx \frac{\left[\chi \left(f,T+\frac{\Delta T}{2}\right)-\chi \left(f,T-\frac{\Delta T}{2}\right)\right]}{\Delta T}.$$

The dielectric data were recorded for temperatures T recorded in steps of 2 °C. For calculating the finite differences at each T, we use ΔT  = 0.05 °C in Equation (15); for this very small ΔT, we assume that TTS is valid and use the HN parameters determined for T to determine χ(f,T±ΔT/2), except for $\tau_{\alpha }$ which was determined from the VFT fit to the BDS data.

## 4. Conclusions

In this article, the molecular relaxations of the isotropic and nematic phases of an acrylate-based LCE, and their response to applied strain, have been determined using a combination of Broadband Dielectric Spectroscopy (BDS), Differential Scanning Calorimetry (DSC), Small Amplitude Oscillatory Shear Rheology (SAOS) and tensile Dynamic Mechanical Analysis (DMA). Due to its lack of phase transitions across a wide *T*-range, once polymerized, our chosen LCE constitutes an excellent model system for investigating the effects of nematic order on the glass-transition-related molecular relaxation behavior. Moreover, when strained, our LCE deforms via a mechanical Fréedericksz transition, demonstrating a molecular auxetic response, whose detailed origin is not well understood [18,19]. To better understand this behavior, we have investigated the effects of deformation on both the relaxation dynamics and the mechanical response of our nematic LCE. 

For the quiescent LCEs, we demonstrate that both the isotropic and nematic LCE samples show a similar T-dependence of their characteristic α relaxation time-scales $\tau_{\alpha }\left(T\right)$ near the glass transition temperatures $T_{g}$; this is reflected in the similar $T_{g}$-values ($T_{g}$ = 279 K and 278 K, as probed by DSC) and dynamic fragilities (D=5.4 and D=5.1; m= 110 and m= 130), for the two phases, which are both representing fragile glass-formers with fragility values consistent with those of polymers [23,24]. Importantly, for both phases, $\tau_{\alpha }\left(T\right)$ qualitatively changes T-dependence at a crossover $T^{*}\approx$ 333 K. For $T>T^{*}$, $\tau_{\alpha }\left(T\right)$ of the nematic LCE is well described as Arrhenius, whereas the isotropic LCE is non-Arrhenius, but more Arrhenius-like (less fragile) above, than below, $T^{*}$. A similar change from non-Arrhenius to Arrhenius at a crossover temperature is often observed for liquid crystal side-chain polymers with nematic phases [42,46].

For comparison, a change in the T-dependence of $\tau_{\alpha }\left(T\right)$ is typically observed in glass-formers at a temperature $T_{B}\sim 1.2-1.6\times T_{g}$ [30], which is comparable to the $T^{*}\sim T_{g}$, observed for our LCEs. However, for non-polymeric non-LC glass-formers, $\tau_{\alpha }\left(T\right)$ is typically less Arrhenius-like (more fragile) above, than below, $T_{B}$ and the change in T-dependence often disappears all together for polymeric systems [31]. Thus, the behavior observed for many nematic systems displays both similarities and clear differences to that of non-LC systems. For our LCE, we stress that the change of phase does not significantly affect the value of $T^{*}$, but it strongly affects how Arrhenius-like the T-dependence of $\tau_{\alpha }\left(T\right)$ is for $T>T^{*}$. We propose that these observations could be related to the existence of pre-transitional phenomena in the form of nematic regions in our ‘isotropic’ LCE, which are locked in by the polymerization. We estimate the size of these regions to be ~1 nm, and demonstrate, by analysis of our BDS data on the isotropic LCE, that correlated segmental motions on similar length-scales are involved in the α relaxation near the crossover at $T^{*}$. 

The extent of decoupling between the ionic conductivity and the structural α relaxation was investigated and we found that the ionic conductivity was significantly more decoupled from the α relaxation in the nematic than in the isotropic LCE. We suggest that differences in dynamic heterogeneity in the two LCE phases could be an important contributing factor in these results. Furthermore, the structural anisotropy induced by the nematic order could in itself play a role in driving the observed decoupling. In addition to the  α relaxation, two further dielectrically active relaxations (β and γ) are observed in both LCE phases. They both demonstrate Arrhenius behavior within the glassy state and are not significantly affected by the LCE phase; we assign these to motions of the mesogen side-chain around its long axis (β), and motions of the alkyl spacer (γ), respectively [22,40]. In addition, a rheology investigation of the isotropic LCE provided evidence for both a Rouse-like mode contribution on time-scales slower than the α relaxation, and an additional relaxation contribution suggested to be due either to free chains moving within the elastomer network, or to the motion of pendant network chains [85,86,87].

Finally, to investigate the origin of molecular auxetic behavior in the nematic LCE, the effects of the applied strain are determined. The complex Young’s modulus, $E^{*}$, and the α relaxation time $\tau_{\alpha }$ are measured for varying applied strains using BDS and DMA. We find that $E^{\prime }$, $E^{\prime \prime }$, and $\tau_{\alpha }\left(T\right)$ remain relatively unchanged for increasing strain, $\epsilon_{t}$, until $\epsilon_{t}>0.55$, where both metrics increase; we associate this increase with a strain-induced increase of constraints on the polymer backbone and the finite extensibility of the network [9,105,109]. Importantly, the mechanical Fréedericksz transition, and the molecular auxetic response, both occur near the region of applied strain, where $E^{\prime }$, $E^{\prime \prime }$, and $\tau_{\alpha }$ increase. Based on the previous suggestion that the molecular auxetic response is linked to the emergence of biaxial order, in turn caused by out-of-plane rotations of mesogenic units (above an applied threshold strain) [19], we suggest that the rotation of the mesogen units is itself driven by the growth of constraints imposed by the increasing strain.

## Figures and Tables

**Table 1:** Results of Arrhenius and VFT fits to relaxations in the isotropic and LCE nematic samples.

Sample	Process	$\tau_{0\;}\left(s\right)$	$\Delta E_{A}\;\left(kJ\;mol^{-1}\right)$	D	$T_{0\;}\left(K\right)$	$T_{g}\;\left(K\right)$
Isotropic	α	$5.3\times 10^{-11}$	-	5.0	243	286
	β	$2.0\times 10^{-19}$	67.9	-	-	-
	γ	$9.3\times 10^{-15}$	29.3	-	-	-
Nematic	α	$1.5\times 10^{-12}$	-	5.6	243	285
	β	$2.1\times 10^{-18}$	63.6	-	-	-
	γ	$7.2\times 10^{-15}$	29.4	-	-	-

**Table 2:** Results of the VFT and Arrhenius fits for the isotropic and nematic LCE.

Sample	1000/T	$\tau_{0}\;\left(s\right)$	$\Delta E_{a}\;\left(kJ\;mol^{-1}\right)$	D	$T_{0}\;\left(K\right)$	$T_{g}\;\left(K\right)$
Isotropic	<3.0	$5.0\times 10^{-14}$	-	16.6	183	270
	>3.0	$2.5\times 10^{-11}$	-	5.4	241	286
Nematic	<3.0	$2.7\times 10^{-16}$	-	17.2	193	275
	<3.0	$1.15\times 10^{-29}$	151.0	-	-	-
	>3.0	$3.8\times 10^{-12}$	-	5.1	245	285

**Table 3:** Results for the VFT-fits of the SAOS data for the isotropic LCE sample.

$\tau_{0}\;\left(s\right)$	D	$T_{0\;}\left(K\right)$	$T_{g}\;\left(K\right)$
$1.33\times 10^{-15}$	8.3	227	276

**Table 4:** Chemical names and mol% for the precursor mixtures of the isotropic and nematic LCEs.

Chemical Name	Mol% of Monomer Mixture
A6OCB	14.6
RM82	7.1
6OCB	55.9
EHA	20.9
MBF	1.5

## Data Availability

Data made available at https://doi.org/10.5518/1074 (latest accessed on 29 November 2021).

## Supplementary Materials

The following are available online, Figure S1: Full dielectric spectra for the isotropic and nematic LCE as a function of temperature, Figure S2: Dielectric spectra in the dielectric modulus and derivative representation, Figure S3: Van Gurp–Palmen graphs for the shear rheology and DMA, Figure S4: Unshifted shear rheology data as a function of temperature, Figure S5: Unshifted DMA data as a function of temperature, Figure S6: DSC and m-DSC traces on the isotropic and nematic LCE samples, Figure S7: normalised BDS $\varepsilon^{\prime \prime }$ data taken at 23 °C as a function of applied true strain for the low and high strain sample.

## Sample Availability

Not available.
