# Supplementary material for: Influence of Liquid Crystallinity and Mechanical Deformation on the Molecular Relaxations of an Auxetic Liquid Crystal Elastomer
Source: Molecules. 2021 Dec 2;26(23):7313. doi: 10.3390/molecules26237313 (PMC8659252; doi:10.3390/molecules26237313)
Supplement: Supplementary file 1 [file molecules-26-07313-s001.zip › molecules-1471271-supplementary.pdf]

molecules

Supplementary Material: Influence of liquid crystallinity and mechanical deformation on the molecular relaxations of an auxetic liquid crystal elastomer

Thomas Raistrick[1], Matthew Reynolds[1], Helen F. Gleeson [1] and Johan Mattsson[1,*]

School of Physics and Astronomy, University of Leeds, Leeds LS2 9JT

Broadband Dielectric Relaxation spectroscopy

Figure S1 shows the dielectric loss ε'' versus frequency f from Broadband Dielectric Spectroscopy (BDS). Three dielectric relaxations are observed, here labelled α, β and γ, as described in detail in the main paper. Data for the isotropic LCE are shown in the left column of Fig. S1 and data for the nematic LCE are shown in the right column. The high temperature data for both the nematic and isotropic LCE samples show DC conductivity characterised by a power-law exponent of -1. To investigate the possible existence of molecular relaxation slower than the structural α-relaxation, the data are investigated both using a dielectric loss modulus (M'') representation and an approach whereby ε' is converted to the dielectric loss (ε''_{der}), as shown in Eq. S1. ε''_{der} provides an approximation of the conduction-free dielectric loss, [1]

$$\varepsilon''_{der}(\omega) = -\frac{\pi}{2}\frac{\partial \varepsilon'(\omega)}{\partial \ln \omega} \text{ where } \omega = 2\pi f. \qquad \text{(eq.S1)}$$

The results for both the M'' and ε''_{der} representation of the BDS data are shown in Fig. S2(a) for T=373.15 K for the nematic LCE. In both the ε''_{der} and M'' representation a peak is observed in the loss spectra, which is not clearly present in the ε'' spectra. Figure S2(b) shows a comparison between the M'' spectra and ε' spectra of the nematic LCE from 373.15 K to 343.15 K in 10 K steps. It is clear that the peaks in the M'' spectra occur in the same frequency range where electrode polarisation is evident by the increase in the ε' spectra. Based on these findings, we interpret the observed contribution as a 'conductivity relaxation' [1], and not as evidence for the presence of a molecular relaxation.

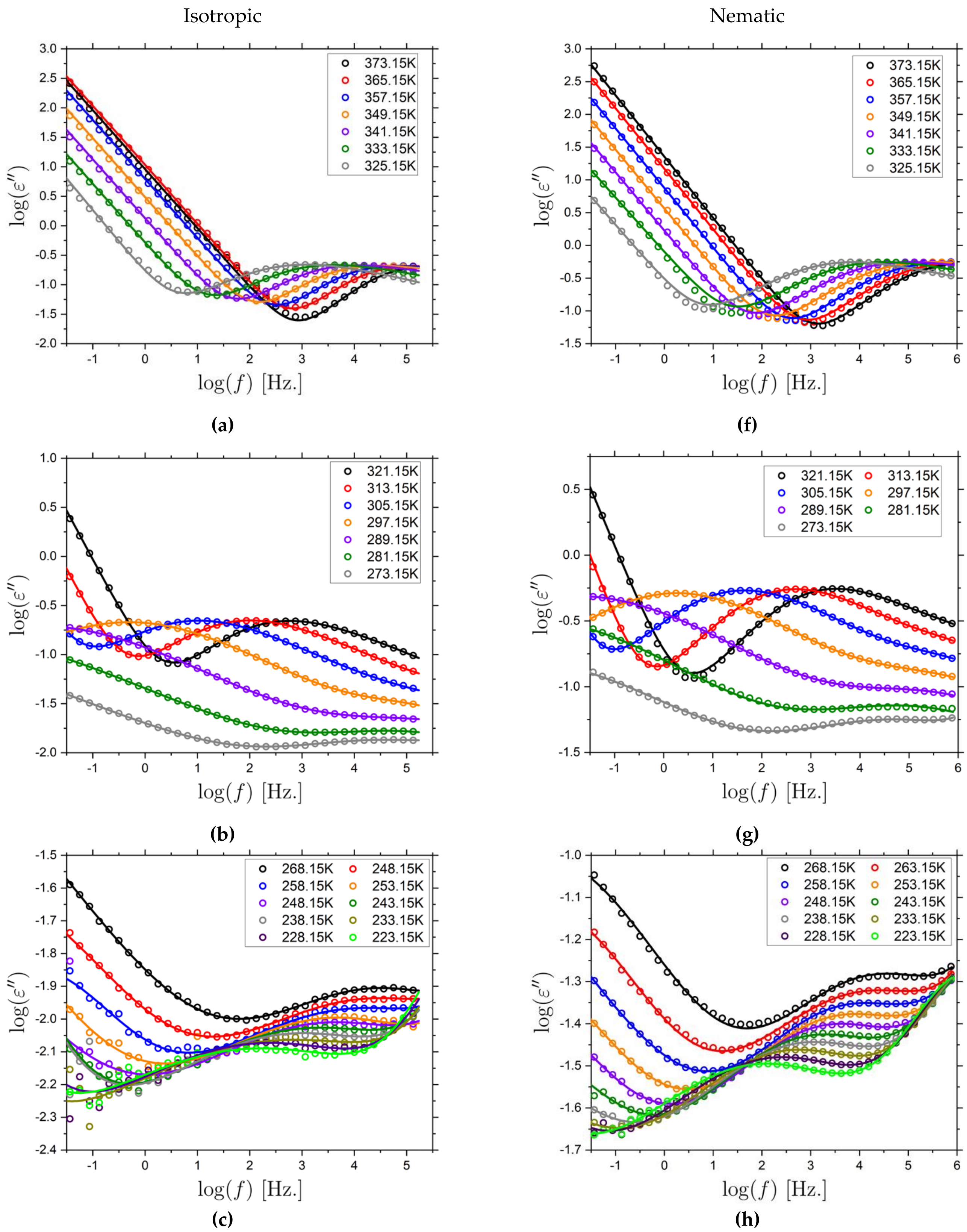

Figure S1. Continued on the next page.

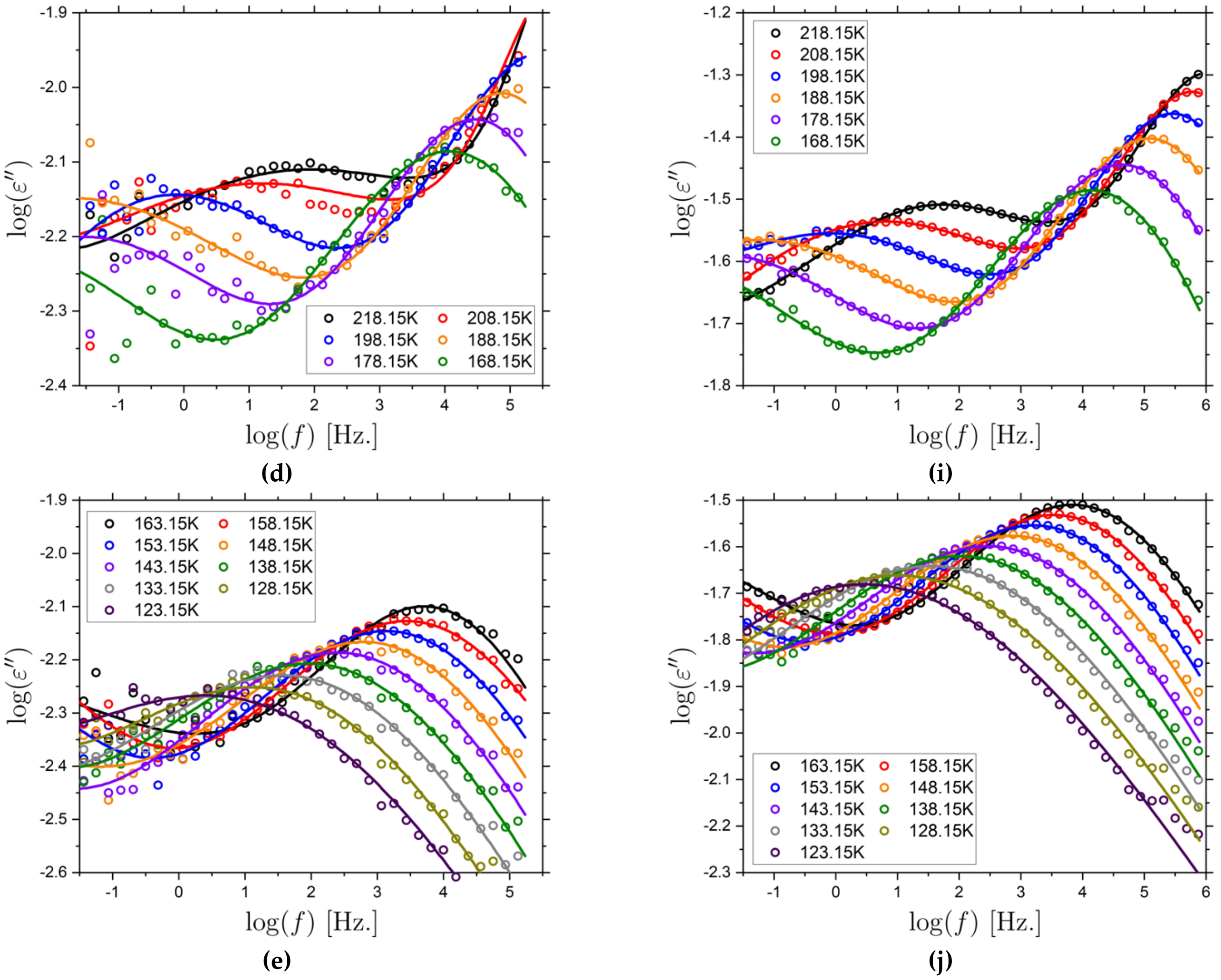

Figure S1. Dielectric loss, ε″, versus frequency, *f*, for the isotropic (left column, a-e) and nematic (right column, f-j) LCE sample.

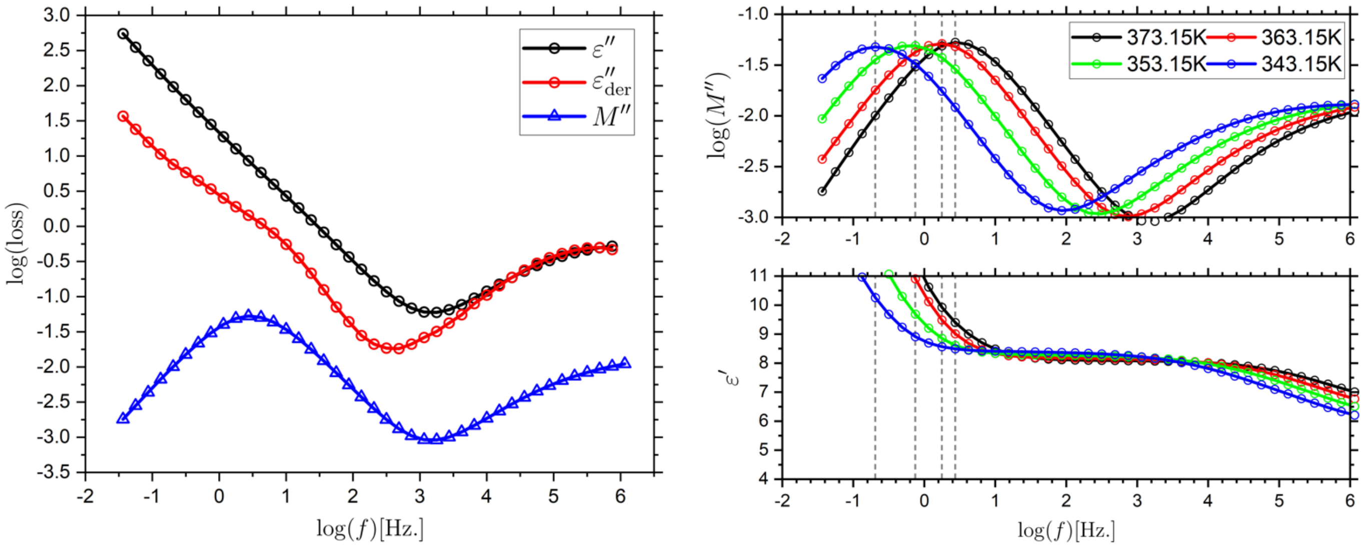

(a) (b)

Figure S2. (a) Dielectric loss spectra at T=273.15K for the nematic LCE in the ε'', ε''_{der} and M'' representation. (b) M'' and ε' versus frequency f for T= 373.15K – 343.15K

Differential Scanning Calorimetry

Differential Scanning Calorimetry (DSC) and modulated DSC (m-DSC) was used to investigate the glass transition of the isotropic and nematic LCE samples and hence the α relaxation. Standard DSC is performed at a rate of 10K/min; this rate typically corresponds to a time-scale $\tau_\alpha \approx 100$ s. [2] On the other hand, m-DSC is performed for a temperature modulation period of 60s, which corresponds to $\tau_\alpha \approx 9.56$s. The corresponding DSC and m-DSC traces are shown in Figure S3.

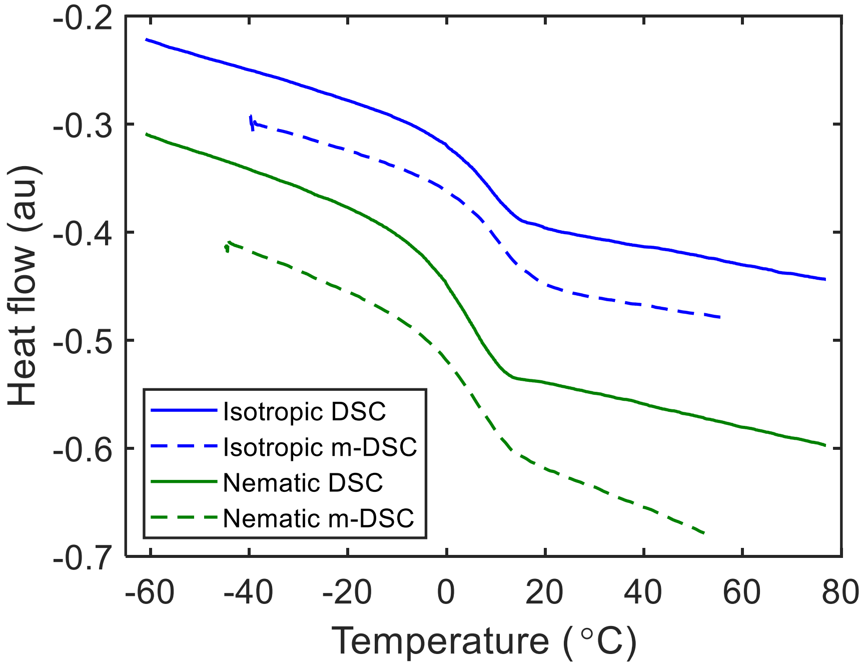

Figure S3. DSC and m-DSC traces for the isotropic and nematic LCE samples.

Rheological investigations

Time Temperature Superposition (TTS) was employed for both the Small Amplitude Oscillatory Shear (SAOS) and Dynamic Mechanical Analysis (DMA) data for the isotropic LCE. For TTS to work, the sample needs to be well approximated as 'thermoreologically simple' meaning that all relaxations follow the same temperature dependence. [3] To investigate the validity of TTS we present the rheology data in so-called van Gurp-Palmen (vGP) plots, [4] where the phase angles (δ) are plotted against the absolute value of the complex shear moduli ($|G^*|$); this representation removes the explicit time-dependence from the data and thus demonstrates whether it is possible to perform TTS based on a frequency shift. Figure S4(a) shows vGP plots for SAOS and DMA data for the isotropic LCE; the complex modulus amplitude is $|G^*|$ and $|E^*|$ for the SAOS and DMA data, respectively. We find that for both SAOS and DMA data, the VGP analysis suggests that frequency-shift-based TTS is a good approximation. To further compare the data from SAOS and DMA, we provide the data in Fig. S4(b) where the DMA data have been shifted for comparison. Fig. S5 and S6 show the unshifted $|G^*|$ and $|E^*|$ data along with $\tan\delta$ for SAOS and DMA, respectively.

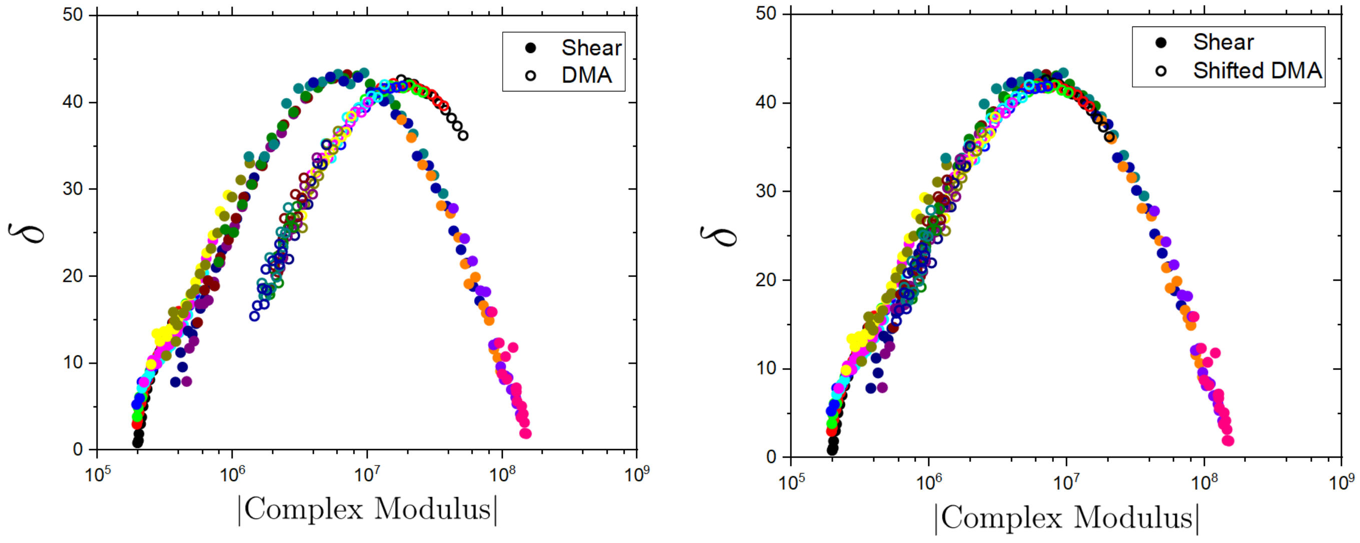

(**a**) (**b**)

Figure S4. (a) Van Gurp-Palmen plots for the SAOS and DMA data. (b) Van Gurp-Palmen plot where the DMA data have been horizontally shifted for comparison.

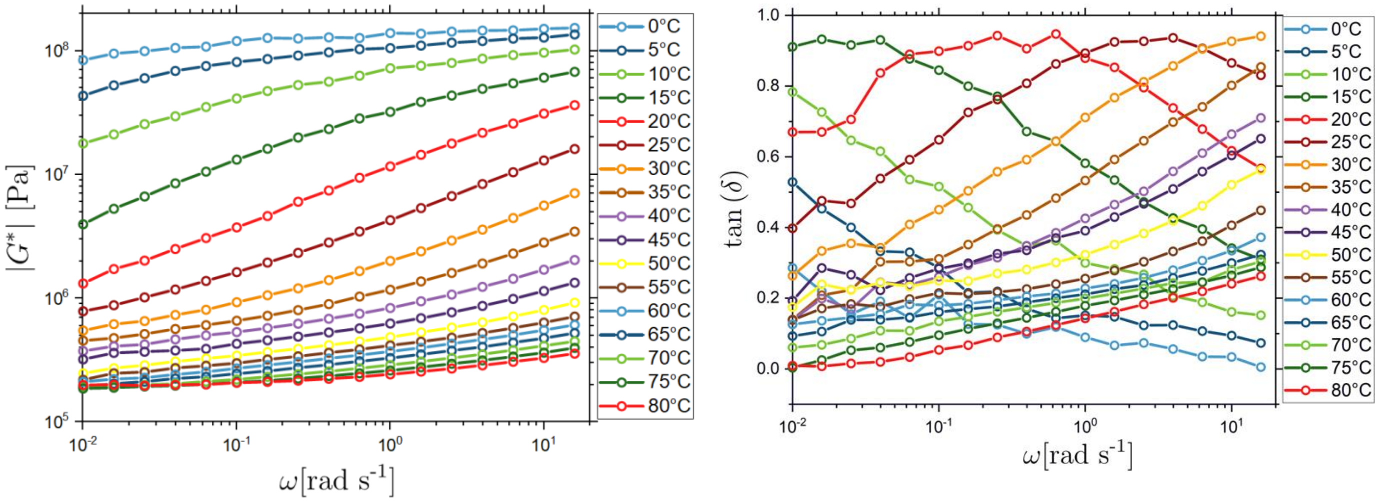

(**a**) (**b**)

Figure S5. Unshifted shear rheology data for different temperatures: (a) amplitude of the complex shear modulus $|G^*|$, and (b) $\tan(\delta)$ versus angular frequency, ω [rad s-1].

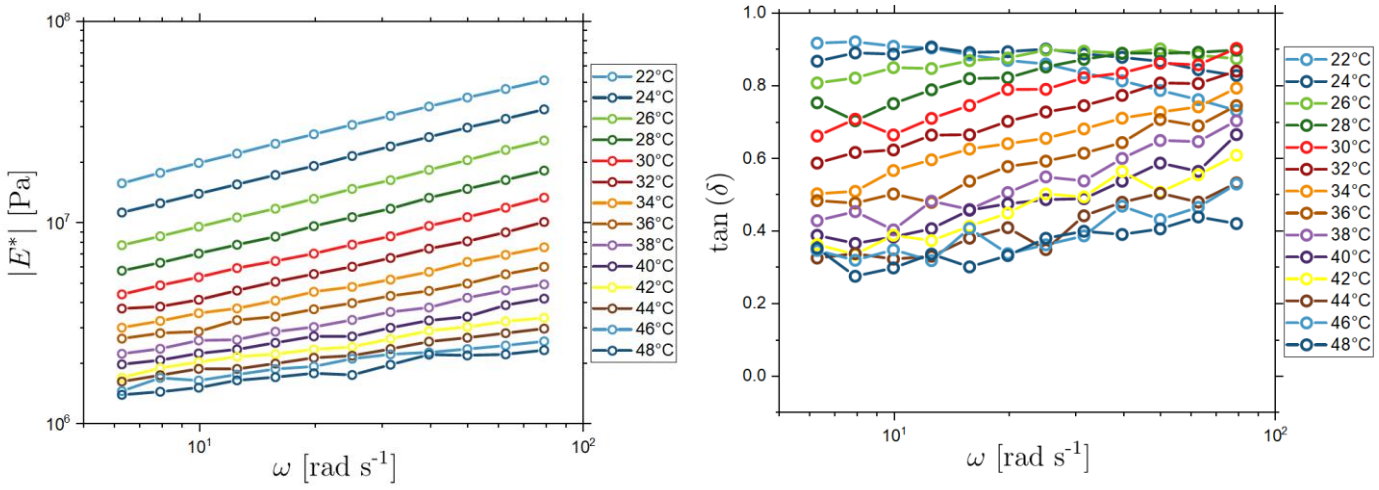

(**a**) (**b**)

Figure S6. Unshifted DMA data for different temperatures: (a) amplitude of the complex Young's modulus $|E^*|$, and (b) $\tan(\delta)$ versus angular frequency, ω [rad s-1].

<u>Broadband Dielectric Relaxation spectroscopy results for strained nematic LCEs</u>

In the main paper BDS results for our nematic LCE are presented as a function of applied strain. Two separate LCE samples were investigated to determine the effect of strain on the α relaxation; one LCE sample was taken to smaller strain values ($\epsilon_t \leq 0.44$) (open triangles), and one to larger strain values ($\epsilon_t \leq 0.86$). Due to slight differences in the unstrained α relaxation timescales between the two samples, the $\tau_\alpha(\epsilon_t)$ data were normalized by the unstrained timescale ($\tau_\alpha(\epsilon_t = 0)$ in the main paper. For clarity, Fig S7 shows the data and the corresponding fits to the data (Fig. S7 a and c) and the resulting α relaxation time-scales without any applied normalisation (Fig. S7 b and d) for both investigated LCE samples.

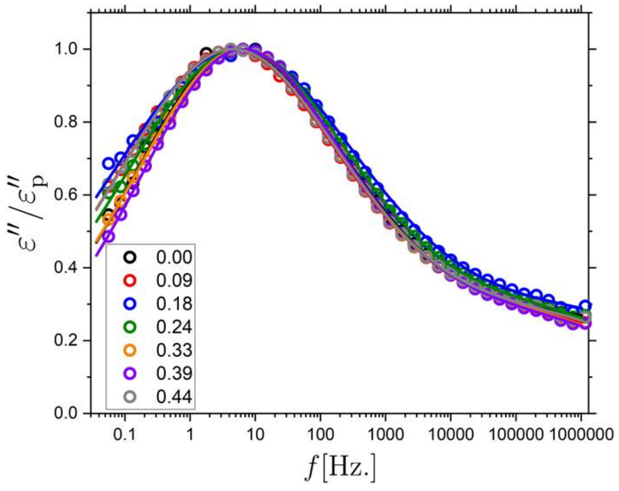

(**a**)

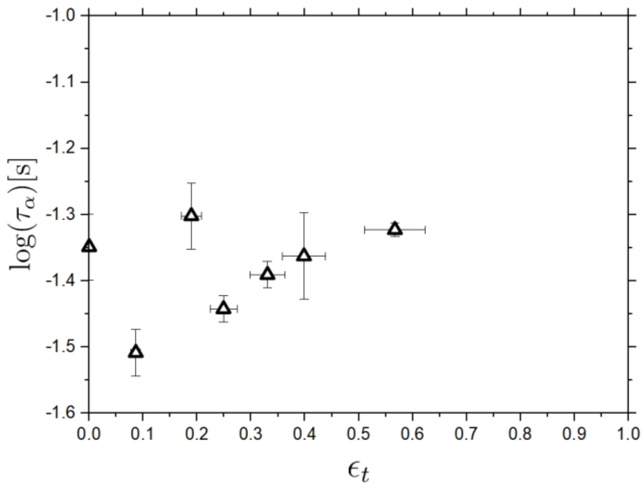

(**b**)

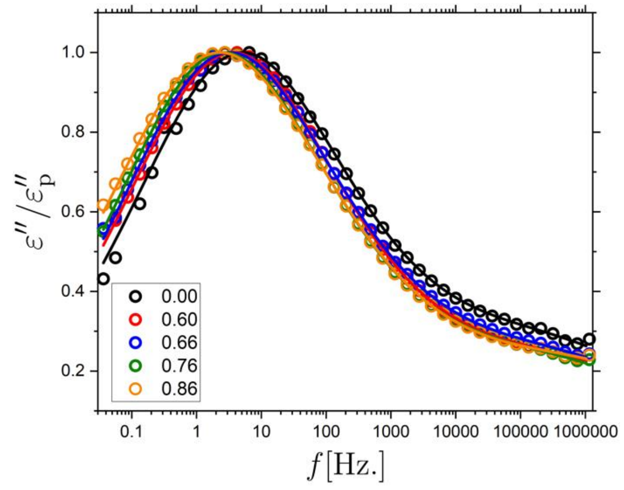

(**c**)

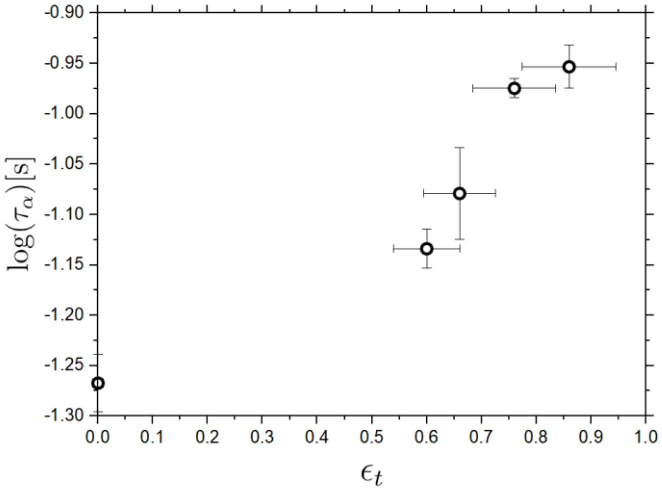

(**d**)

Figure S7. (a) Normalised BDS loss data taken at T=23°C as a function of applied true strain (ϵ_t) ranging from 0.00 to 0.44. (b) the corresponding α relaxation time as a function of ϵ_t from 0.00 to 0.44. (c) ε'' vs frequency for true strain (ϵ_t) from 0.00 to 0.86. (b) corresponding relaxation time scale for the α relaxation as a function of true strain from 0.00 to 0.86.

1. Wübbenhorst, M.; van Turnhout, J. Analysis of Complex Dielectric Spectra. I. One-Dimensional Derivative Techniques and Three-Dimensional Modelling. *Journal of Non-Crystalline Solids* **2002**, *305*, 40–49, doi:10.1016/S0022-3093(02)01086-4.
2. Hempel, E.; Hempel, G.; Hensel, A.; Schick, C.; Donth, E. Characteristic Length of Dynamic Glass Transition near T_g for a Wide Assortment of Glass-Forming Substances. *J. Phys. Chem. B* **2000**, *104*, 2460–2466, doi:10.1021/jp991153f.
3. Plazek, D.J. 1995 Bingham Medal Address: Oh, Thermorheological Simplicity, Wherefore Art Thou? *Journal of Rheology* **1996**, *40*, 987–1014, doi:10.1122/1.550776.
4. van Gurp, M.; Palmen, J. Time-Temperature Superposition For Polymeric Blends. *Rheology Bulletin* **1998**, *67*, 5–8.
